# Age-Related Variations in Regional White Matter Volumetry and Microstructure During the Post-adolescence Period: A Cross-Sectional Study of a Cohort of 1,713 University Students

**DOI:** 10.3389/fnsys.2021.692152

**Published:** 2021-08-03

**Authors:** Ami Tsuchida, Alexandre Laurent, Fabrice Crivello, Laurent Petit, Antonietta Pepe, Naka Beguedou, Stephanie Debette, Christophe Tzourio, Bernard Mazoyer

**Affiliations:** ^1^Groupe d’Imagerie Neurofonctionnelle, Institut des Maladies Neurodégénératives, UMR 5293, Université de Bordeaux, Bordeaux, France; ^2^Groupe d’Imagerie Neurofonctionnelle, Institut des Maladies Neurodégénératives, UMR 5293, CNRS, Bordeaux, France; ^3^Groupe d’Imagerie Neurofonctionnelle, Institut des Maladies Neurodégénératives, UMR 5293, CEA, Bordeaux, France; ^4^Université de Bordeaux, Inserm, Bordeaux Population Health Research Center, U1219, CHU Bordeaux, Bordeaux, France; ^5^Centre Hospitalier Universitaire, Bordeaux, France

**Keywords:** MRI, diffusion, white matter, DTI, NODDI, post-adolescence, cohort, cross-sectional

## Abstract

Human brain white matter undergoes a protracted maturation that continues well into adulthood. Recent advances in diffusion-weighted imaging (DWI) methods allow detailed characterizations of the microstructural architecture of white matter, and they are increasingly utilized to study white matter changes during development and aging. However, relatively little is known about the late maturational changes in the microstructural architecture of white matter during post-adolescence. Here we report on regional changes in white matter volume and microstructure in young adults undergoing university-level education. As part of the MRi-Share multi-modal brain MRI database, multi-shell, high angular resolution DWI data were acquired in a unique sample of 1,713 university students aged 18–26. We assessed the age and sex dependence of diffusion metrics derived from diffusion tensor imaging (DTI) and neurite orientation dispersion and density imaging (NODDI) in the white matter regions as defined in the John Hopkins University (JHU) white matter labels atlas. We demonstrate that while regional white matter volume is relatively stable over the age range of our sample, the white matter microstructural properties show clear age-related variations. Globally, it is characterized by a robust increase in neurite density index (NDI), and to a lesser extent, orientation dispersion index (ODI). These changes are accompanied by a decrease in diffusivity. In contrast, there is minimal age-related variation in fractional anisotropy. There are regional variations in these microstructural changes: some tracts, most notably cingulum bundles, show a strong age-related increase in NDI coupled with decreases in radial and mean diffusivity, while others, mainly cortico-spinal projection tracts, primarily show an ODI increase and axial diffusivity decrease. These age-related variations are not different between males and females, but males show higher NDI and ODI and lower diffusivity than females across many tracts. These findings emphasize the complexity of changes in white matter structure occurring in this critical period of late maturation in early adulthood.

## Introduction

Early adulthood is characterized by significant changes in lifestyle and behavior for many, when individuals explore their identity and various life possibilities to become fully independent. For some, it involves the attainment of higher education and training to acquire new skills and knowledge necessary for their planned vocation. Although the most dramatic development in the human brain takes place earlier in life, with the total brain volume reaching 90% of the adult volume by the age of 5 years (Dekaban, 1978; Lenroot and Giedd, 2006), both global and regional changes in brain structure and function persist throughout childhood and adolescence, and some of the maturational changes continue well into adulthood (Dumontheil, 2016). In particular, the white matter (WM) of the brain shows a protracted course of development, with its total volume continuing to increase up to the fourth or fifth decade of life (Walhovd et al., 2011; Lebel et al., 2012). The development of WM microstructure is also sensitive to common life experiences in young adults, including exposure to alcohol and tobacco, and other recreational drugs (Bava et al., 2013; Gogliettino et al., 2016; Silveri et al., 2016), changes in sleep patterns (Elvsåshagen et al., 2015; Telzer et al., 2015), and intensive motor and cognitive training (Scholz et al., 2009; Lövdén et al., 2010; Mackey et al., 2012; Schlegel et al., 2012; Lakhani et al., 2016). Detailed characterization of the late maturational processes of the WM in young adults is crucial for elucidating how the learning and other life experiences may shape the structural and functional organization of the brain through their impact on the brain wiring. Understanding the normative development during this period may also shed light on the vulnerability of this particular period in life to various neuropsychiatric disorders, such as substance abuse, mood and anxiety disorders (Kessler et al., 2007).

What we know about normative WM development primarily comes from non-invasive neuroimaging of typically developing individuals with magnetic resonance imaging (MRI). In addition to the macro-structural changes that can be measured with T1-weighted images, diffusion-weighted imaging (DWI) methods allow detailed characterizations of the WM microstructural properties. Over the past two decades, studies using DWI have provided much insight into the WM microstructural changes during development (reviewed in Lebel and Deoni, 2018; Tamnes et al., 2018; Lebel et al., 2019). The majority of these studies quantify DWI through a diffusion tensor imaging (DTI) model representing the direction and magnitude of diffusion of tissue water molecules as a single tensor in each voxel (Tournier et al., 2011). Most commonly, fractional anisotropy (FA), which measures the degree of diffusion directionality, is used to quantify maturational changes, with an increase in FA attributed to myelination and increased axonal size or packing. Other DTI measures include axial and radial diffusivity (AD/RD), representing diffusion along the longest and shortest axis, respectively, of the tensor modeled in each voxel, and mean diffusivity (MD), representing the average magnitude of diffusion. Across studies, FA increases and overall decreases in diffusivity with increasing age are observed in most WM regions through childhood and adolescence (e.g., Bonekamp et al., 2007; Lebel et al., 2008; Giorgio et al., 2010; Tamnes et al., 2010; Lebel and Beaulieu, 2011; Brouwer et al., 2012; Simmonds et al., 2014; Pohl et al., 2016). In a large-scale, multi-cohort study, we have recently demonstrated that such changes continue up to early to mid-adulthood (Beaudet et al., 2020).

However, being a “signal” based model, the DTI model only describes the diffusion process in each voxel and does not attempt to delineate signals attributable to different biological tissue components (Ferizi et al., 2017). Thus, changes in DTI metrics only indicate alterations in magnitude or directionality of diffusivity, and different biological processes that affect diffusion properties of the tissue cannot be distinguished (Jones et al., 2013). More concretely, FA can be increased due to myelination or increased axonal packing but would decrease with increasing fiber population complexity (e.g., crossing fibers). In contrast, “tissue” based models attempt to estimate the components of underlying tissue, typically using DWI acquisitions with multiple *b*-values, and likely provide more biologically specific insights (Alexander et al., 2019). One such model is neurite orientation dispersion and density imaging (NODDI), which models three tissue compartments (intra- and extra-cellular and cerebrospinal fluid). It estimates separate indices for neurite density (neurite density index, NDI) and fiber orientation complexity (orientation dispersion index, ODI), together with the isotropic volume fraction (i.e., cerebrospinal fluid compartment, IsoVF) (Zhang et al., 2012). Several recent studies have used NODDI to examine developmental changes in the WM microstructural properties through infancy (Jelescu et al., 2015; Dean et al., 2017), childhood to adolescence (Genc et al., 2017; Mah et al., 2017; Dimond et al., 2020; Lynch et al., 2020). These studies have indicated an age-related increase in NDI, with very little change observed in ODI in the first two decades of life (Mah et al., 2017; Dimond et al., 2020; Lynch et al., 2020), although studies covering a wider age range indicate that ODI in many WM tracts starts to increase in early adulthood (Chang et al., 2015; Slater et al., 2019). Nevertheless, a large-scale study focusing on the period of early adulthood to detail the late maturational changes in regional WM properties is still lacking.

In the present study, we characterize variations in WM-related metrics, including regional volumes and microstructural properties measured using both DTI and NODDI, in the MRiShare database, a large cross-sectional cohort of young adults undergoing university-level education (Tsuchida et al., 2020). This study’s primary goal is to document the age-related variations in the regional WM properties in this cohort. We also report on the interrelations among the age effects on different WM metrics in an effort to better understand biophysical processes underlying the late maturational changes in the WM. The secondary goal is to gain much-needed insights into the sexual dimorphism of developmental processes (Lebel et al., 2019) by investigating the effects of sex on these WM metrics and their age-related variations.

## Materials and Methods

### Participants

The MRi-Share study protocol was approved by the local ethics committee (CPP2015-A00850-49). All participants were recruited through the larger i-Share cohort study (for internet-based Student Health Research enterprise).^1^ Participants signed an informed written consent form and received compensation for their contribution. Out of 2,000 individuals who were enrolled between October 2015 and June 2017, 1,823 completed the MRI acquisition protocol for both structural (T1-weighted and FLAIR) and diffusion imaging. While the study protocol allowed enrollment of students up to 35 years of age, almost 95% of our sample was under 26 years old. In this study, we present the estimated age effect on WM metrics in the sub-sample of participants aged 18–26 (mean ± SD = 21.7 ± 1.8 years, *N* = 1,713). Age distribution was similar in males (mean ± SD = 21.9 ± 1.8 years, *N* = 467) and females (mean ± SD = 21.7 ± 1.7 years, *N* = 1,246), with only a marginal difference in their mean (2 months difference in age, *p* = 0.066, Welch’s *t-*test). The higher proportion of females relative to males in MRi-Share is a feature observed among university students at the French national level that is amplified in the i-Share cohort due to an over-recruitment of students coming from faculties in which an even greater proportion of women are observed.

### MRI Acquisition

The complete MRi-Share brain imaging acquisition and analysis protocols of the MRi-Share study have been detailed in Tsuchida et al. (2020). Briefly, all MRI data were acquired on the same Siemens 3T Prisma scanner with a 64-channels head coil (gradients: 80 mT/m–200 T/m/s) in the 2 years between November 2015 and November 2017. The MRi-Share acquisition protocol closely emulated that of the UKB MR brain imaging study (Alfaro-Almagro et al., 2018), in terms of both modalities and scanning parameters, with the exception of task-related functional MRI that was not acquired in MRi-Share participants. Here, we will focus on the MRi-Share structural (T1 and T2-FLAIR) and DWI brain imaging protocol. The key acquisition parameters for these scans were as follows;

- T1-weighted sagittal 3D-MPRAGE [repetition time (TR)/echo time (TE)/inversion time (TI) = 2,000/2.0/880 ms, in-plane acceleration factor (R) = 2, spatial resolution = 1 × 1 × 1 mm^3^ isotropic, matrix size = 192 × 256 × 256, duration = 4 min 54 s].
- T2-weighted sagittal 3D-SPACE-FLAIR [TR/TE/TI = 5,000/394.0/1,800 ms, *R* = 2, partial Fourier (PF) = 7/8, spatial resolution = 1 × 1 × 1 mm^3^ isotropic, matrix size = 192 × 256 × 256, duration = 5 min 50 s].
- 2D axial DWI (multi-band factor = 3, TR/TE = 3,540/75.0 ms, R = 1, PF = 6/8, fat-saturation, spatial resolution = 1.75 × 1.75 × 1.75 mm^3^ isotropic, matrix size = 118 × 118 × 84, duration = 9 min 45 s).

For the DWI we acquired 8, 32, and 64 directions each for *b*-values 300, 1,000, and 2,000 s/mm^2^, respectively, and acquired eight pairs of *b* = 0 images acquired in Anterior-Posterior (AP) and the reverse PA phase encoding, interleaved during the *b* > 0 acquisition. The spatial resolution of the DWI was 1.75 × 1.75 × 1.75 mm^3^ isotropic, which was slightly better than that of UKB (2 × 2 × 2 mm^3^ isotropic).

### Image Processing

The acquired images were managed and processed with the Automated Brain Anatomy for Cohort Imaging platform (ABACI, IDDN.FR.001.410013.000.S.P.2016.000.31235; details in Tsuchida et al., 2020). Below we briefly describe the processing steps in each pipeline pertaining to the generation of the JHU atlas ROI image-derived phenotypes presented in the current paper.

#### T1 and T2-FLAIR Structural Pipeline

Our structural pipeline processed T1 and FLAIR images for multi-channel volume- and surface-based morphometry, primarily with SPM12^2^ and Freesurfer v6.0.^3^ For generating the regional WM volumes based on JHU atlas, we used the Jacobian-modulated WM probability map (1 mm isotropic) outputted by the “Unified Segmentation” framework (Ashburner and Friston, 2005) in the SPM-based volume processing branch of our pipeline (for details, see Tsuchida et al., 2020). The same Jacobian-modulated WM map was also used to obtain the total WM volume (TWMV). We also obtained the total intracranial volume (TIV) estimate based on the Freesurfer-branch of our pipeline.

#### Field Map Generation Pipeline

As in the UKB (Alfaro-Almagro et al., 2018), we estimated the fieldmap images from the *b* = 0 images with opposing AP-PA phase-encoding directions from DWI scans rather than from “traditional” fieldmaps based on dual echo-time gradient-echo images. We used all eight pairs of AP/PA *b* = 0 images that were interspersed in the DWI scan to estimate the susceptibility induced field and motion across the interspersed *b* = 0 scans using the topup tool (Andersson et al., 2003) from the FMRIB Software Library (FSL, v5.0.10).^4^ The resulting subject motion parameters and the estimate of susceptibility induced off-resonance field were passed to the DWI pipeline. It also generated the brain mask based on the average distortion-corrected b0 maps, also used for the distortion corrections in the DWI pipeline.

#### Diffusion MRI Pipeline

A detailed description of the preprocessing steps of DWI is provided by Tsuchida et al. (2020). Briefly, the DWI data were first corrected for susceptibility and eddy-current distortion using the FSL Eddy tool, with replacement of outlier slices (*eddy_openmp* as implemented in FSL v5.0.10 patch; Andersson et al., 2016; Andersson and Sotiropoulos, 2016) and denoised by applying non-local means filter using “*nlmeans*” denoising tool (Coupe et al., 2008, 2011) as implemented in the *Dipy* package (0.12.0; Garyfallidis et al., 2014).^5^ The resulting image was then used to fit (1) DTI (Diffusion-Tensor Imaging; Basser et al., 1994) modeling and (2) microstructural model fitting with NODDI (Neurite Orientation Dispersion and Density Imaging; Zhang et al., 2012). For fitting DTI, volumes with the highest *b*-value (*b* = 2,000 s/mm^2^) were stripped from the data, as the accuracy of the fit starts to decrease above *b* = 1,000 s/mm^2^ (Jensen and Helpern, 2010). Note that it still used multi-shell data, using volumes with both *b* = 300 and 1,000 s/mm^2^ in addition to *b* = 0 images. The diffusivity maps were further cleaned by removing diffusivity value outliers using Random Sample Consensus (RANSAC) approach (Choi et al., 2009), as implemented in the *scikit-learn* package (0.19.1).^6^ The denoising, DTI computation, and the RANSAC outlier removal were performed by wrapping *Scipy* scripts, developed by Sherbrooke Connectivity Imaging Lab.^7^ For NODDI, the full set of multi-shell data was used for the fitting. We also used the empirical values of cohort-specific isotropic and parallel diffusivity as the dPar and dIso parameters for fitting NODDI (set to 1.5 × 10^–3^ and 2.4 × 10^–3^ mm^2^/s, respectively), which were obtained by computing the mean MD within lateral ventricles and mean AD within the corpus callosum in individual T1 space for each subject. The preprocessing and DTI fitting were performed using tools from FSL and the *Dipy* package, while the AMICO (Accelerated Microstructure Imaging via Convex Optimization) tool (Daducci et al., 2015) was used for NODDI fitting. For each participant, the DWI processing pipeline produced seven images in native space: fractional anisotropy (FA), mean, axial, and radial diffusivity (MD, AD, and RD), based on DTI modeling, neurite density index (NDI), orientation dispersion index (ODI), and isotropic volume fraction (IsoVF), derived from NODDI.

#### Generation of JHU Atlas Region WM Phenotypes

**FIGURE 1:**
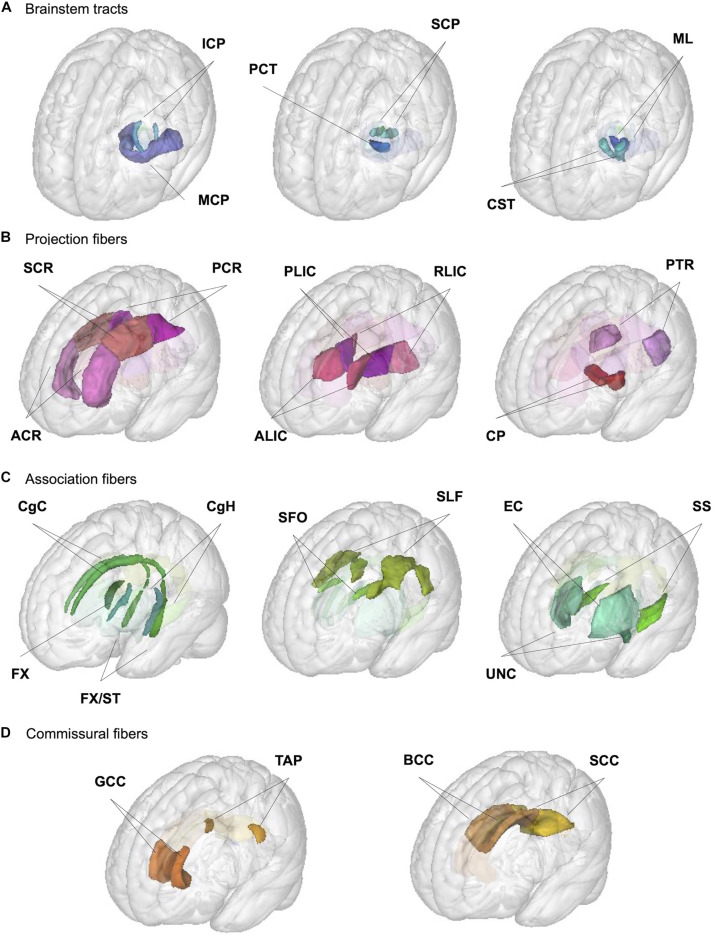
Illustration of JHU ROIs used in the analysis. Locations of the 27 ROIs (6 medially located plus 21 pairs of ROIs in each hemisphere) from the JHU ICBM-DTI-81 white matter labels atlas are shown in the glass brain for each broad group; **(A)** brainstem, **(B)** projection, **(C)** association, and **(D)** commissural fibers. See Table 1 for the full ROI name corresponding to the abbreviations in the figure.

We used the JHU ICBM-DTI-81 white matter labels atlas (Mori et al., 2008; Oishi et al., 2008) to generate regional phenotypes for each of the following metrics: regional WM volume and mean values for 4 DTI (FA, MD, AD, and RD) and 3 NODDI (NDI, ODI, and IsoVF) metrics. We used the atlas packaged with FSL v5.0.10, which does not have the orientation or labeling issues noted in other versions (Rohlfing, 2013) but is missing medial longitudinal fasciculus and inferior fronto-occipital fasciculus ROIs described by the authors of the atlas (Mori et al., 2008). We extracted the WM volume and mean DTI/NODDI values for 48 ROIs in this atlas, but in the absence of strong evidence for the hemispheric asymmetry in the age-related changes (Lebel and Beaulieu, 2011; Slater et al., 2019; Dimond et al., 2020), we combined values across the right and left hemispheres for the 21 pairs of ROIs present in each hemisphere by taking the average between the pair of ROIs, which were weighted by the respective volumes of each ROI in the case of DTI/NODDI metrics, to reduce the number of comparisons. Table 1 provides the abbreviations of ROIs used in the figures and tables throughout the manuscript, and Figure 1 presents the locations of these ROIs. They are organized according to the broad classification used by the author of the atlas: (1) tracts in the brainstem, (2) projection fibers, (3) association fibers, and (4) commissural fibers (Mori et al., 2008).

**TABLE 1:** Abbreviations of JHU atlas ROI names.

ROI name	Abbreviation	Hemisphere side	ROI name	Abbreviation	Hemisphere side

**Brainstem**			**Association**		
Middle cerebellar peduncle	MCP	Both	Fornix	FX	Both
Pontine crossing tract	PCT	Both	Fornix cres or stria terminalis	FX/ST	Right/Left
Corticospinal tract	CST	Right/Left	Cingulum cingulate gyrus	CgC	Right/Left
Medial lemniscus	ML	Right/Left	Cingulum hippocampus	CgH	Right/Left
Superior cerebellar peduncle	SCP	Right/Left	Superior fronto-occipital fasciculus	SFO	Right/Left
Inferior cerebellar peduncle	ICP	Right/Left	Superior longitudinal fasciculus	SLF	Right/Left
**Projection**			External capsule	EC	Right/Left
Anterior corona radiata	ACR	Right/Left	Uncinate fasciculus	UNC	Right/Left
Superior corona radiata	SCR	Right/Left	Sagittal stratum	SS	Right/Left
Posterior corona radiata	PCR	Right/Left	**Commissural**		
Anterior limb of the internal capsule	ALIC	Right/Left	Genu corpus callosum	GCC	Both
Posterior limb of the internal capsule	PLIC	Right/Left	Body corpus callosum	BCC	Both
Retrolenticular part of the internal capsule	RLIC	Right/Left	Splenium corpus callosum	SCC	Both
Posterior thalamic radiation	PTR	Right/Left	Tapetum	TAP	Right/Left
Cerebral peduncle	CP	Right/Left			

**FIGURE 2:**
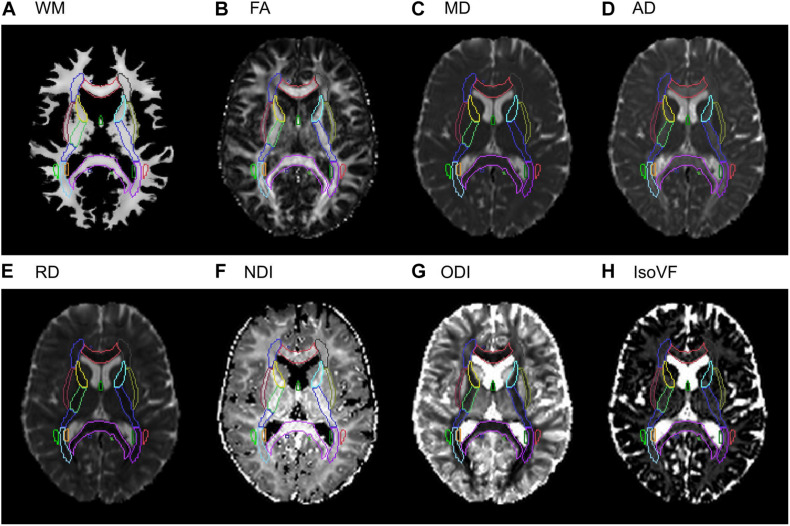
Examples of the WM tissue map and DTI/NODDI maps from a representative subject in the stereotaxic space. A selected axial slice from **(A)** the Jacobian-modulated WM tissue map, **(B)** FA, **(C)** MD, **(D)** AD, **(E)** RD, **(F)** NDI, **(G)** ODI, **(H)** IsoVF maps in the stereotaxic space is shown for a representative subject. Outlines of the JHU ICBM-DTI-81 white matter labels atlas are shown for each image to show the quality of the alignment.

For extracting the regional DTI and NODDI values, we first computed the rigid transform for aligning DTI and NODDI maps to the native T1 reference space (1 × 1 × 1 mm^3^ isotropic) with the SPM12 “Coregister” function. This transform was then aggregated with the deformation field generated in the structural pipeline to transform DTI/NODDI maps in the native DWI space to the standard template space in one step, using the SPM12 “Normalize” function. When computing the mean values within each of the 48 ROIs, we used the subject-specific, spatially normalized WM probability map, thresholded at 0.5, as an inclusive mask. It ensured that the mean values were computed within regions that are primarily WM, and minimized the partial volume effects from the surrounding non-WM tissues. Figure 2 provides the example images of WM tissue map and DTI/NODDI maps from a representative subject, with the outlines of JHU ROIs to show the quality of alignment.

### Quality Control

A detailed description of the quality control (QC) procedure for image analysis is provided in Tsuchida et al. (2020). Briefly, all structural scans were reviewed by one of the three experienced MD investigators of the MRiShare study to check for major artifacts or structural abnormalities before processing. During image processing of the structural or DWI pipelines, pipeline-specific QC images were generated for each subject. For the structural pipeline that generated reference T1 images for other modalities, a trained rater (N.B.) reviewed individual subject-specific QC images for each step of the processing for all subjects and verified that the quality of the SPM-based tissue segmentation and spatial normalization were satisfactory. For the DWI pipeline, a number of subject-specific QC images and quantifiable QC metrics mainly related to the quality of DWI data were generated (see Supplementary Material). Additional QC metrics for the spatial normalization were extracted by computing the image similarity of individual WM tissue probability map and DTI and NODDI scalar maps to the cohort-average maps, using Fisher *z*-transformed Pearson’s correlation *r* between the two images. Two investigators (A.T. and L.P.) identified and reviewed the subject-specific QC images for those with extreme values in any of the QC metrics, but none of them showed any obvious signs of noticeable problems in the raw DWI or the scalar DTI and NODDI maps and their spatial normalization, except in a few cases where midsagittal plots of the raw DWI revealed a zig-zag pattern indicative of the within-volume motion in a few volumes.

Similarly, we checked the group-level distributions at the level of individual phenotypes for any missing values and the extreme outliers. Four subjects did not have any volumetric or DTI/NODDI values for fornix (FX), as the WM probability map did not overlap with this small ROI in the standard space. For the same reason, one subject was missing data for the tapetum (TAP). In addition, for corticospinal tract (CST; *n* = 4) and inferior cerebellar peduncle (ICP; *n* = 6) ROIs, mean DTI/NODDI values were not computed in the pipeline since these ROIs extended beyond the bounding box of the DWI-derived images in the standard space. Beyond these missing data, the extreme outliers were rare, and each phenotype was roughly normally distributed. Exceptions were some ROIs, in particular those surrounded by cerebrospinal fluid and/or relatively small ROIs (e.g., FX, TAP, brainstem ROIs), which had slightly skewed distributions, most likely caused by slight misalignments in DWI-derived images and structural images in standard space.

We checked for the impact of both phenotypic and QC metric outliers by removing the “far out” outliers (Tukey, 1977), defined as those with values below or above three times interquartile range (IQR) from the first or third quartile, respectively, for either the individual phenotype or any of the quantitative QC metrics. In addition to the phenotypic and QC metric outlier removal, we investigated the effect of including a global image quality metric as a covariate in the model. For the WM volume, we used the Euler number computed by Freesurfer that has been shown to be consistently correlated with the manual rating of the quality of the structural image (Rosen et al., 2018). For the DWI-based metrics, we used the mean relative RMS of the volume to volume displacement that quantifies the in-scanner motion since a recent study has demonstrated that both DTI and NODDI mean values were impacted by this QC metric (Pines et al., 2020). However, the effects of outliers or inclusion of these global quality metrics on the analyses were relatively minor (see Supplementary Material). For simplicity, here we report the results without any outlier removal, with total sample size of 1,713 for all ROI-metric combinations, except for FX (*N* = 1,709), TAP (*N* = 1,712), CST (*N* = 1,709), and ICP (*N* = 1,707) ROIs.

### Statistical Analysis

The primary goal of the present manuscript is to describe the age-related variations in the regional WM volumes and microstructural properties in young adults. Although not our primary focus, we included sex as a covariate, and report the global pattern, mainly to characterize any overall differences between the two sexes at this age range and to examine any sex dependency in the observed age effects by including age by sex interaction term. Given our sample’s narrow target age range, we expected most of the age-related variations in the volumetric and diffusion metrics to be captured by a linear age model. Indeed, the inspection of raw scatter plots (see Supplementary Material) did not suggest any ROIs showing any clear non-linear patterns of age-dependency. Also, a preliminary comparison of models with and without quadratic age effect to capture any non-linear trend showed that linear age effect models were sufficient for each metric and ROI combinations, as judged by the Bayesian information criterion (BIC; data not shown). Thus, for all metrics, we tested the following model;

Y∼α+βAgeAge+βSexSex+βAgeAge×Sex×Sex

We also checked the consistency of the reported age effect estimates on the regional WM volumes when correcting for the global volume (TIV), and in the case of the DTI/NODDI metrics, examined the effects of correcting for both the global (TIV) and regional (ROI) volumes, and report them in Supplementary Material.

In an effort to better understand biophysical processes underlying the late maturational changes in the WM, we performed an exploratory analysis of the interrelations among the age effect estimates of the WM metrics. For this, we first computed the standardized parameter estimates (β^∗^) of the age effect for each of the eight WM metrics across the 27 ROIs, and calculated pairwise Pearson’s correlations between the β^∗^ values in the 27 ROIs for given metrics (e.g., FA vs. NDI, NDI vs. ODI, etc.). Note that it quantifies the correlation between the estimates of age effects across the 27 ROIs, and not the raw correlations between metric values in the ROIs, although perfect correlations in the underlying raw data would result in the perfect correlations in the estimated effects of age and sex as well. That is, if two metrics measure a single property of WM and are perfectly correlated, the age or sex effect estimates for such hypothetical metrics would also be perfectly correlated. In reality, if two metrics represent related but distinct properties that are differentially sensitive to age or sex, correlation structures for the respective effects would be different. To illustrate this point, we also present a similar correlation structure for the estimated mean values in the ROIs across the sexes (to account for the fact that the age effect estimates also represent the value across both sexes) using the metric values standardized across the ROIs.

All model fits were performed in R, version 3.4.4 (R Core Team, 2018). We used the *lm* function as implemented in the stats library for fitting the model. The goodness of fit was assessed with adjusted *R*^2^. The Sex contrast was deviation-coded using “contr.sum” setting so that parameter estimate (β) and *t* statistics for non-categorical variables (i.e., age in our case) represent those across sexes, and not for the specific reference sex (as would be in the case of treatment-coding, in the presence of interaction terms). Age was mean-centerd so that the intercept represented the value at group mean age. For all analyses, we report *p*-values as significant when below 0.05 after Bonferroni correction for multiple tests (27 ROIs × 8 measures, nominal *p* threshold = 0.05/216 = 0.00023). We also report generalized eta squared (η^2^*_*G*_*) as a measure of effect size (Olejnik and Algina, 2003), obtained using *aov_car* function in *afex* package (Singmann et al., 2021), including all terms in the model as the “observed” variables. The specification of the observed variables (as opposed to manipulated variables in other research designs) allows the correction of the effect size estimate, which makes this measure less dependent on specific research design features (Olejnik and Algina, 2003).

Visualizations of statistical summaries were created with *ggplot2* (Wickham, 2016), and tables were created with the *gt* package (Iannone et al., 2020) in R. Linear fitting of age effects for each sex was performed by predicting the given WM property in each sex using the *emmeans* package (Lenth, 2021). For evaluating the interrelations between the age-related variations in the regional WM volumes and diffusion metrics, we first computed the β*^∗^* values for the respective terms in each metric using the robust standardization through refitting, implemented with the *effectsize* package (Ben-Shachar et al., 2020). Then, the β*^∗^* values across 27 ROIs were used to compute Pearson’s correlation between the pairwise metrics. The computation of correlation values and visualization of the results was performed using the *Ggally* package (Schloerke et al., 2021).

## Results

### The Main Effect of Age

**FIGURE 3:**
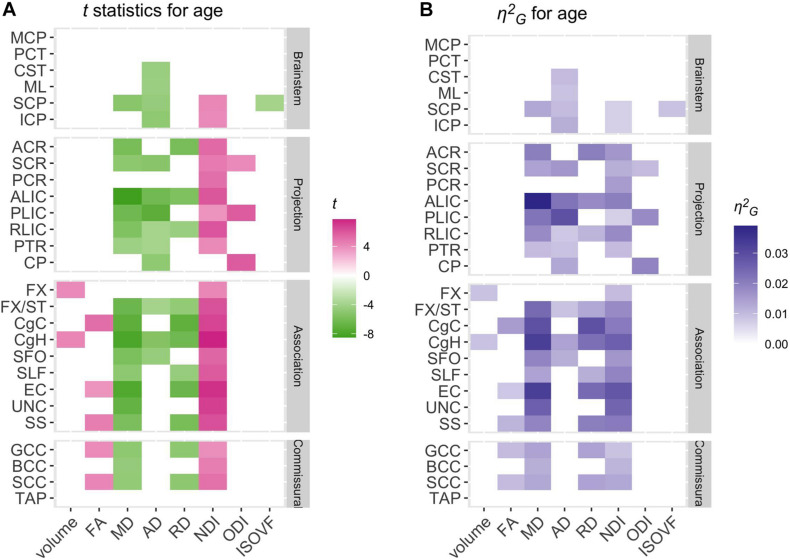
Patterns of significant age effects across WM volume and diffusion phenotypes and ROIs. Relative statistical strengths and effect sizes of age effects across diffusion phenotypes and ROIs are shown as heatmaps of **(A)** *t* statistics and **(B)** η^2^_*G*_ values (see Table 1 for the full names for the abbreviated ROIs). Those that did not survive Bonferroni corrections for multiple comparisons were filtered out (set to 0) to facilitate comparisons within significant results. Positive *t-*scores in pink indicate an age-related increase and negative values in green indicate an age-related decrease.

**FIGURE 4:**
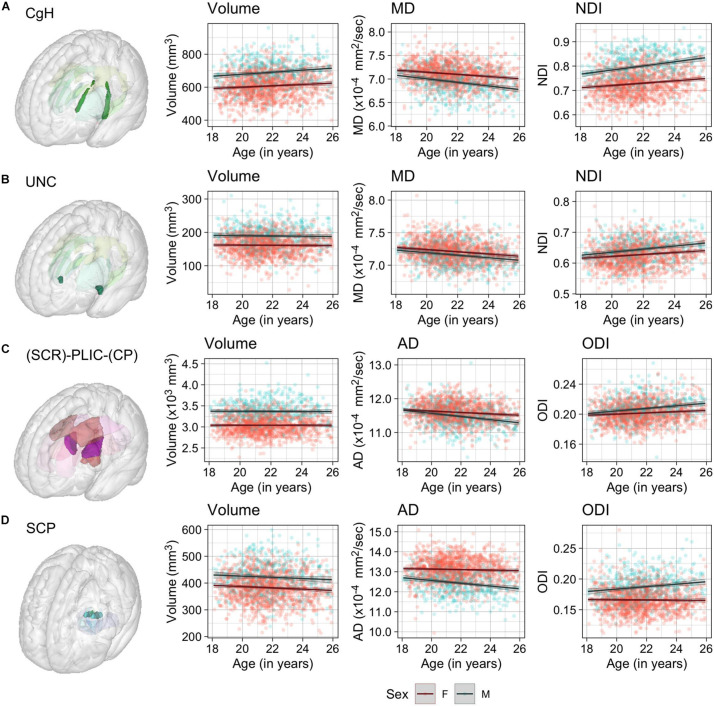
Scatter plots of individual age effects in **(A)** the cingulum-hippocampus (CgH), **(B)** uncinate fasciculus (UNC), **(C)** posterior limb of the internal capsule (PLIC), and **(D)** superior cerebellar peduncle (SCP) ROIs. Predicted linear regression lines are superimposed for each sex (dark red: females, dark cyan: males), with shades indicating the 95% confidence intervals.

Table 2 presents the parameter estimates (β) for age effects for each metric (WM volume, 4 DTI and 3 NODDI metrics) across the ROIs, and Figure 3 visually presents the summary by showing the *t* statistics and effect sizes (η^2^*_*G*_*) as heatmaps, filtering out those that did not survive Bonferroni corrections. Supplementary Tables 1–8 provide the complete model results, including the confidence intervals of age β, uncorrected *p-*values and η^2^*_*G*_*, and total variance explained by the model for each metric and ROI. Figure 4 provides selected scatter plots of age effects for each sex to present examples of such effects. Similar plots of age effects for the entire metrics and ROIs are also provided in Supplementary Figures 5–12. As evident in Table 2 and Figure 3, a number of WM ROIs showed robust age-related variations in one or more metrics we examined.

**TABLE 2:** Summary of age effects for each diffusion phenotype across JHU ROIs.

	Volume	FA (×10^–3^)	MD (×10^–6^)	AD (×10^–6^)	RD (×10^–6^)	NDI (×10^–3^)	ODI (×10^–3^)	IsoVF (×10^–3^)
**Brainstem**								

MCP	–25.2	–0.0	−0.9*	−1.4*	–0.6	1.1*	0.7*	−0.6*
PCT	–2.7	–0.7	–1.9	−3.3*	–1.2	0.3	1.3*	−1.9*
CST	–1.8	−1.3*	−2.6*	−5.6***	–1.1	0.2	1.9*	−2.0*
ML	–1.2	–1.1	−2.4*	−5.1***	–1.1	2.2*	1.3	−1.5*
SCP	−2.3*	–0.3	−2.0***	−4.1***	−0.9*	2.0***	0.9*	−1.2***
ICP	–0.4	–0.3	−2.1**	−3.6***	−1.4*	2.4***	1.1*	−1.2*

**Projection**								

ACR	3.2	1.4**	−2.0***	−1.4*	−2.3***	2.9***	–0.2	–0.1
SCR	5.6	–0.6	−1.3***	−2.6***	−0.6*	2.2***	1.2***	–0.1
PCR	3.3	0.6	−1.3**	−1.3*	−1.2*	2.8***	0.4	0.5*
ALIC	1.7	0.6	−2.0***	−2.8***	−1.6***	3.1***	0.7*	–0.4
PLIC	–0.5	−0.7*	−1.4***	−3.4***	–0.5	1.8**	1.3***	−0.6**
RLIC	–0.9	0.8*	−1.6***	−1.8**	−1.5***	3.4***	0.5*	–0.1
PTR	–1.4	0.3	−1.4***	−2.2**	−1.0*	2.1***	0.5*	0.1
CP	–3.9	−1.2*	−2.2*	−5.8***	–0.4	1.4	2.4***	−1.6*

**Association**								

FX	2.6***	0.7	0.3	1.1	–0.1	3.5***	1.4*	2.0*
FX/ST	–0.2	0.9*	−2.2***	−3.0***	−1.7***	3.7***	0.8*	−0.6*
CgC	8.3**	2.3***	−2.0***	–0.5	−2.7***	3.8***	–0.5	–0.3
CgH	5.1***	1.2*	−3.0***	−3.6***	−2.8***	6.6***	1.6**	–0.2
SFO	0.5	0.1	−1.8***	−2.8***	−1.3*	3.5***	0.6	–0.1
SLF	6.3	1.0*	−1.3***	–0.8	−1.5***	2.6***	–0.1	0.1
EC	3.8	1.2**	−1.7***	−1.4**	−1.9***	3.3***	0.3	0.2
UNC	–0.3	1.0	−1.8***	−2.1*	−1.7**	4.1***	0.8*	0.7*
SS	–0.8	1.6***	−2.0***	−1.2*	−2.4***	3.9***	–0.0	0.1

**Commissural**								

GCC	5.1	1.7***	−1.7***	–0.8	−2.1***	2.4***	–0.4	–0.3
BCC	21.7	0.7	−1.3***	−1.5*	−1.2**	2.4***	0.3	–0.1
SCC	31.5*	1.4***	−1.5***	–1.0	−1.7***	2.6***	0.1	−0.5*
TAP	−2.7*	2.0*	−1.4*	0.5	−2.4*	2.3*	–0.3	0.5

Non-standardized parameter estimates (β) for age effects for each phenotype and ROI are shown (see Table 1 for the full names of abbreviated ROIs). The unit of the age effect is mm^3^/year for the volume, mm^2^/s/year for the diffusivity measures (MD, AD, and RD), and/year for FA and the NODDI phenotypes (NDI, ODI, IsoVF). Statistical significance symbols (uncorrected for multiple comparisons) *0.05 < p < 0.001, **0.001 < p < 0.0001, ***p < 0.0001. Bold symbols indicate Bonferroni-corrected significant p-values.

Significant age-related increases in WM volumes were observed only in cingulum hippocampus (CgH) and fornix (FX). The cingulum in the cingulate gyrus (CgC) showed a significant age-related increase when TIV or TWMV was accounted for by including them in the model (see Supplementary Material).

In contrast, robust age effects in DTI and NODDI metrics were observed across many ROIs, most pronounced for MD and NDI (Figure 3). Those with significant age effects all showed an age-related increase in NDI, and decreases in diffusivity metrics. Many of these ROIs showed a tendency for the volumetric increase as well, but some showed a significant NDI increase and diffusivity decrease without any trend for volumetric increase (see Figure 4 for examples in CgH, with the volumetric increase, and uncinate fasciculus (UNC), without). CgH additionally showed a significant age-related decrease in AD and a trend for an ODI increase. The AD decrease was also observed across many ROIs in projection fibers and brainstem ROIs with varying degrees but was particularly pronounced in the ROIs that represent a connected pathway of projection fibers: superior corona radiata (SCR), posterior limb of the internal capsule (PLIC), and cerebral peduncle (CP) (see Figure 4 for example in PLIC), all of which also showed a significant ODI increase with age.

### Interrelations Among the Age Effects on the WM Properties

**FIGURE 5:**
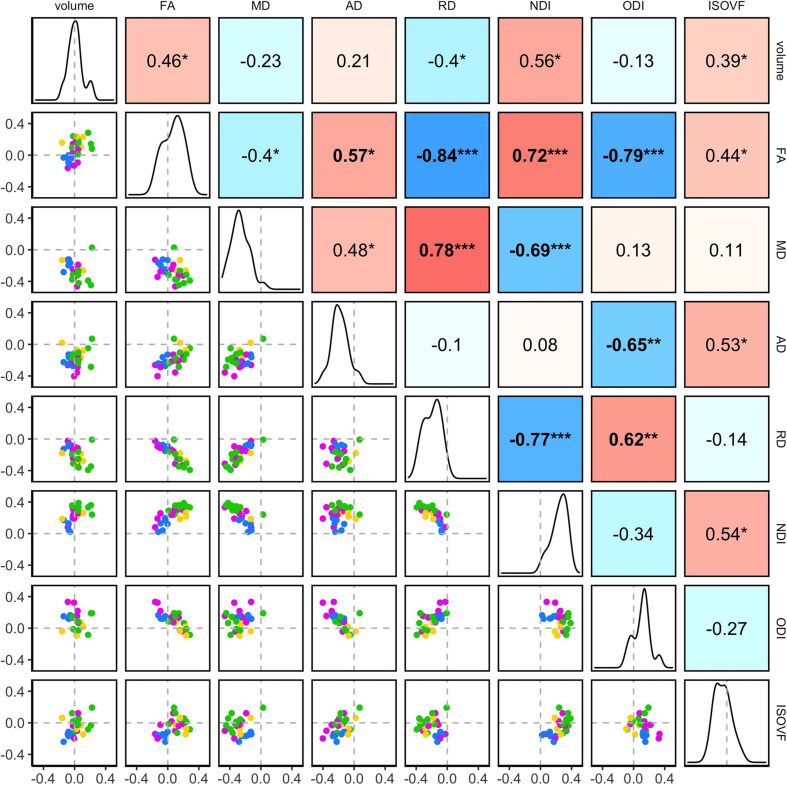
The inter-relations between the age-related variations in the regional WM properties. Pairwise correlations of the standardized parameter estimates (β*) for age effects in the 27 ROIs are shown. The diagonal of the plot matrix shows the distributions of β*_Age_ for the regional WM volume and DTI/NODDI values. The upper triangle shows Pearson’s correlation (*r*) values. The lower triangle shows the pairwise scatter plots of β*_Age_, with the colors indicating the ROI groups (*blue*: brainstem, *pink*: projection, *green*: association, *yellow*: commissural). Statistical significance symbols (uncorrected for multiple comparisons) *0.05 < *p* < 0.001, **0.001 < *p* < 0.0001, ***p < 0.0001. Bold-face indicates a significant correlation after Bonferroni correction for multiple comparisons (28 correlations).

Figure 5 shows the correlation plot of the standardized parameter estimates (β*^∗^*) for the age effect between pairs of metrics across the 27 ROIs. For a comparison, Supplementary Figure 13 shows a similar plot computed for the simple regional mean values of these metrics, calculated after standardizing values across the ROIs.

The correlation structure of the age effect β*^∗^* values indicated that overall, the degree of age-related variations in the regional mean FA values was negatively associated with RD and positively with AD. Thus, although both AD and RD decreased with age across the most ROIs, regions with faster age-related decreases in RD relative to AD showed overall age-related increases in FA. The degree of age-related variations in FA was also negatively associated with ODI. These patterns are expected since FA is, by definition, higher when diffusivity along the axial axis is higher than along the radial axis and when fiber orientation dispersion is lower. Indeed, such patterns were more evident in the correlations of simple mean values of the regional WM metrics, which showed a strong positive correlation between the regional FA and AD values and also strong negative correlations between the regional FA and RD or ODI values.

In contrast, the correlation patterns for NDI were distinct between the regional age effects and the simple mean values: the degree of age-related increases in NDI was positively associated with the degree of age-related variations in the regional WM volume and FA, and negatively associated with the age-related decrease in RD (i.e., regions with more NDI increases showing more volumetric and FA increases and RD decreases). In the regional mean values, the higher NDI values were not strongly associated with the regional WM volumes or FA and RD values. Another difference was the non-significant but negative correlation between the age effects on NDI and ODI, indicating the ROIs showing more age-related increases in NDI tended to show less ODI increases, while in the regional mean values, NDI and ODI were weakly but positively associated, indicating higher NDI values in ROIs with higher ODI. Note that despite the weak correlations between the age effects on the regional volume and FA, RD, and NDI values, the age effects in these microstructural properties were not affected by the inclusion of the regional volumes as a covariate in the model (see Supplementary Material).

### Dependency of Age-Related Variations on Sex

**FIGURE 6:**
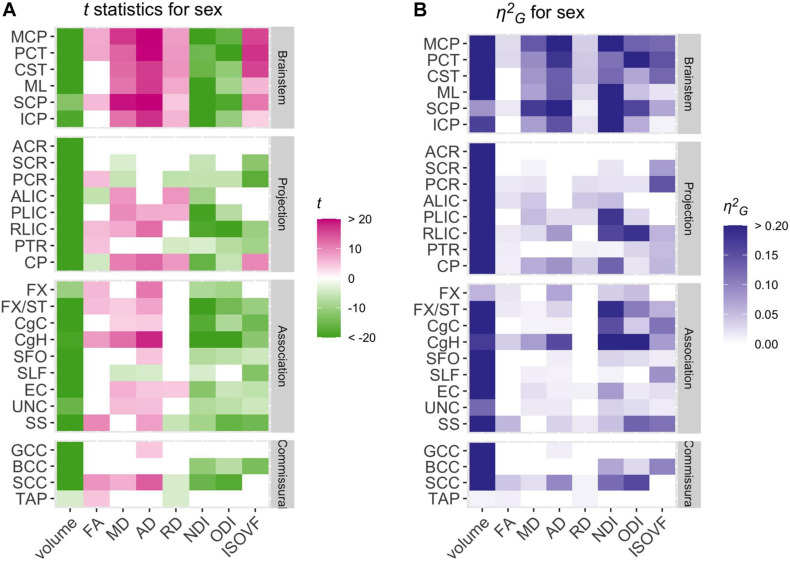
Patterns of significant sex effects across WM volume and diffusion phenotypes and ROIs. Relative statistical strengths and effect sizes of sex effects across WM phenotypes and ROIs are shown as heatmaps of **(A)** *t* statistics and **(B)** η^2^_*G*_ values (see Table 1 for the full names for the abbreviated ROIs). Those that did not survive Bonferroni corrections for multiple comparisons were filtered out (set to 0) to facilitate comparisons within significant results. Positive *t-*scores in pink indicate higher values in females than in males and negative values in green indicate the opposite.

Summary of *t* statistics and η^2^*_*G*_* values for the sex effect on each of the eight WM properties across the JHU ROIs are presented in Figure 6 (see also Supplementary Tables 1–8).

Not surprisingly, males had larger WM volumes than females across most of the ROIs examined. However, the difference diminished considerably when global volume differences were taken into account by including either TIV or TWMV in the model (see Supplementary Material). For the diffusion metrics, females showed higher diffusivity than males across many ROIs, while males showed higher NDI and ODI overall. There were relatively few regionally specific patterns in the sex effects, although the differences were most robust in the brainstem ROIs.

Despite the widespread main effects of sex, we did not observe any significant sex differences in the age-related variations in the WM properties (the lowest uncorrected *p* = 0.0008). Overall, any non-significant sex differences in the age-related trajectory tended to show a steeper slope in males than in females, in particular for AD and ODI (see for example in the SCP, Figure 4 and Supplementary Figures 5–12).

## Discussion

The primary objective of the present study was to characterize the late maturational changes in the regional WM properties during post-adolescence in the large and unique sample from the MRi-Share database. We also examined sex differences in the WM of this sample and assessed whether the age-related changes differed between the two sexes. Below we discuss our main findings in relation to the existing literature, comment on the specific features of our dataset, and methodological strengths and limitations of the present study.

### Age-Related Variations in Regional WM Properties

We observed widespread age-related increases in the NDI as well as decreases in diffusivity (MD, AD, and RD) across many of the JHU ROIs in our sample of young adults aged between 18 and 26 years. Changes in FA were statistically weaker, but ROIs with significant age effects all showed an increase with age. Regional volumes did not vary significantly with age for the most part but showed trends for an age-related increase in some ROIs. The degree of age-related increases in FA and volume in each ROI were nonetheless correlated with the degree of age-related variations in the NDI and diffusivity. Regionally, we observed that many ROIs in projection and brainstem fiber groups showed primarily significant age-related decreases in AD. In contrast, those in association and commissural fiber groups were more characterized by decreases in RD. Several ROIs in the corticospinal pathway additionally showed age-related increases in ODI.

The global patterns we observed in our sample are consistent with a wealth of literature showing a relatively protracted maturation of human brain WM: both developmental and life-span studies of WM volume and DTI metrics have indicated continued increases in global WM volume and FA into young adulthood, together with decreases in diffusivity that peaks sometime in young to mid-adulthood (Hasan et al., 2007, 2010; Westlye et al., 2010; Lebel et al., 2012; Slater et al., 2019; Beaudet et al., 2020; Tsuchida et al., 2020). More recent studies using NODDI have also shown the continuous increase of NDI through development (Genc et al., 2017; Mah et al., 2017; Dimond et al., 2020; Lynch et al., 2020; Pines et al., 2020) and adulthood (Billiet et al., 2015; Chang et al., 2015; but see Kodiweera et al., 2016), peaking around the fourth and fifth decade of life (Slater et al., 2019; Qian et al., 2020). ODI, on the other hand, has not been reported to change noticeably during development (Dimond et al., 2020) or show a slight decrease in some tracts (Lynch et al., 2020) but starts to increase during young adulthood (Chang et al., 2015; Slater et al., 2019) that continues through aging (Billiet et al., 2015; Beck et al., 2021).

More robust and wide-spread increase in NDI than FA observed in our data likely results from the fact that we sampled the FA values from the entire WM regions within each ROI, rather than a limited “core” region with high FA values, a common approach in studies using the same JHU atlas, as discussed in the section on Potential limitations below. When sampling over regions with more complex fiber organizations, NODDI can provide more specific insights than FA, since FA can be influenced by both the fiber density and myelination as well as by the composition of fiber orientations (among other things) in the sampled voxel (Zhang et al., 2012; Jones et al., 2013). This point is corroborated by the relationships we observed between the age effects on the regional FA and NDI or ODI; while the age-related increase in FA was positively correlated with that of NDI, it was negatively correlated with the degree of age-related increase in ODI. It suggests that concomitant increases in NDI and ODI can have an opposing impact on the regional FA, rendering it less sensitive to the effects of age.

Regionally, we observed that cingulum WM showed a prominent age-related increase in NDI as well as MD and RD decreases. With concurrent RD reduction, the NDI increase is suggestive of increased myelination (Song et al., 2005). Cingulum WM in hippocampal region (CgH) also showed a robust volumetric increase as well, both in terms of raw volume and relative to TIV or TWMV. However, in these and other ROIs, the regional volume had little impact on the observed age effects on other WM properties, suggesting the distinct biological processes governing the age-related changes in WM volumes and other metrics related to microstructural properties (Lebel et al., 2019). Previous studies have indicated cingulum to be one of the last major tracts to mature during development, reaching peak values in FA or minimum values in MD later than other tracts (Tamnes et al., 2010; Westlye et al., 2010; Lebel et al., 2012). Similarly, a higher rate of NDI growth in limbic tracts that include CgC and CgH has been reported in a sample of 66 healthy subjects with a mean age of 25 years (Chang et al., 2015). A more recent and larger-scale lifespan study on regional DTI and NODDI metrics in 801 individuals aged 7–84 years has also indicated a relatively late peak age for NDI in CgC and CgH (Slater et al., 2019). The cingulum bundle primarily contains fibers that link cingulate gyrus and hippocampus (Mori et al., 2008), but also consists of many short association fibers that interconnect medial parts of the frontal, parietal, and temporal regions (Heilbronner and Haber, 2014). With the diverse fiber populations that make up this bundle, neuroimaging studies in healthy subjects as well as in clinical populations have implicated this region for a wide range of cognitive functions: these include executive control, motivation, and pain in anterior/dorsal cingulate and memory in hippocampal region (reviewed in Bubb et al., 2018). Several studies have also shown the link between the microstructural integrity of the cingulum bundle and cognitive performance in children (Bathelt et al., 2019) and older adults (Kantarci et al., 2011; Bettcher et al., 2016). In this context, robust age-related changes observed in the cingulum ROIs in our sample of young adults undergoing higher-level education are particularly interesting. Future studies should investigate the relevance of volumetric and microstructural differences across subjects in cingulum to cognitive and academic performance and emotional and behavioral development.

Beyond the cingulum bundle, all association ROIs tended to show a higher increase in NDI (average annual percentage increase, computed from the base value at age 18) of 0.55%/year, ranging from 0.37%/year in SLF to 0.89%/year in CgH) than commissural (average of 0.35%/year, ranging from 0.32%/year in GCC and 0.41%/year in TAP) and projection ROIs (average of 0.34%/year, ranging from 0.16%/year in CP and 0.45%/year in ACR and RLIC). The NDI increase was smallest in the brainstem ROIs (average of 0.16%/year) and least statistically significant. Of note, the brainstem ROIs also had the highest estimated NDI at age 18 [mean (range) = 0.89 (0.84–0.96)], while association fiber ROIs had the lowest estimated NDI at the same age [mean (range) = 0.71 (0.62–0.80)]. It suggests that most of the NDI growth in brainstem ROIs likely takes place earlier than the age range of our sample. The observed pattern is broadly consistent with previous DTI studies suggesting earlier maturation in the commissural and projection fibers, followed by association fibers, especially in fronto-temporal regions (Tamnes et al., 2010; Westlye et al., 2010; Lebel et al., 2012). More recent studies with NODDI also support similar regional patterns of the developmental trajectory (Dean et al., 2017; Slater et al., 2019; Lynch et al., 2020). For instance, in a recent study examining the maturational timing of regional NODDI parameters in a cross-sectional sample of 104 subjects aged between 0 and 18 years, the NDI growth in callosal fibers reached a plateau the earliest, followed by projection and association fibers (Lynch et al., 2020).

While relatively modest in terms of NDI growth, we found that the connected ROIs of projection fibers, from superior corona radiata (SCR), through the posterior limb of the internal capsule (PLIC), then to cerebral peduncle (CP), showed the age-related increase in ODI and decrease in AD. It suggests the increasing fiber complexity in this large WM bundle that contains the pyramidal and cortico-pontine tracts. This observation is novel, and has not been reported in previous studies examining age-related variations in regional NODDI values in subjects with age-range that overlaps with our study (Billiet et al., 2015; Chang et al., 2015; Slater et al., 2019; Pines et al., 2020). None of these studies reported notable age-related ODI increase in this projection fiber pathway that stood out from other regions (e.g., non-brainstem projection fiber ROI in Chang et al., 2015 and tractography-based corticospinal tract in Slater et al., 2019). However, different methodology in defining the tract ROI as well as modeling strategies makes the direct comparison difficult. Future studies are needed to confirm the validity of our observation and investigate the functional relevance of such age-related changes.

### Biophysical Interpretation of Age-Related Variations in DTI and NODDI

The present study demonstrates the usefulness of NODDI metrics in at least partially disambiguating the factors that can result in the observed patterns of age-related variations in DTI metrics: the overall age-related decreases in diffusivity were associated with two uncorrelated increases in NDI and ODI, with NDI increases associated with decreases in RD and ODI with decreases primarily in AD. It indicates that the age-related variations in DTI metrics at this age range likely result from changes in both the intra-neurite fraction and fiber complexity. However, it should be cautioned that as in any other models, NODDI makes certain assumptions that over-simplify the underlying microstructure, and it has been criticized in recent years that some of these assumptions are invalid and can introduce biases in the estimates (Jelescu et al., 2015; Lampinen et al., 2017). In particular, the assumption of a single and fixed intrinsic diffusivity for both intra- and extracellular space that causes non-negligible biases in ODI and IsoVF, as well as large uncertainty in the IsoVF estimation (Jelescu et al., 2015). NDI has also been shown to be overestimated in the tissue with lower diffusivity than assumed in the model, such as in the gray matter and in pathology (Lampinen et al., 2017). Even when the estimates are free from biases, the underlying biological phenomena are not as specific as the naming of NDI (“neurite density” index) suggests, since any microstructural changes that can affect intra-neurite fraction directly (increase in the number and density of axons) or indirectly by affecting the volume of the extra-axonal space (for example myelination). Such ambiguity is evident in a number of speculative interpretations in the clinical applications of NODDI in the literature (Kamiya et al., 2020). Ultimately, precise biological interpretations of observed changes or variations in NODDI should be validated through comparisons with histological studies and with complementary or higher-order diffusion models (Jelescu et al., 2020). Nonetheless, in the case of the white matter in normal development, it is likely that the observed patterns of NODDI and DTI metrics reflect the myelination and remodeling of myelin, rather than an increase in the number of axons (de Graaf-Peters and Hadders-Algra, 2006; Sampaio-Baptista and Johansen-Berg, 2017). The correlation of the age-related increases in NDI with decreases in RD, but not with AD, is consistent with this interpretation. Future studies should investigate the validity of this observation, and also examine how such changes in young adults are affected by cognitive and physical activities, and other lifestyle and environmental factors.

### Sex Differences in the WM Properties and the Patterns of Age-Related Variations

In many ROIs, we detected significant sex differences in the regional WM properties but found very little evidence for sex differences in the age-related variations in the WM properties at this age range. The sex differences in the regional WM volumes were most likely due to differences in overall head size, as the inclusion of TIV or TWMV diminished most of the differences. However, we also observed globally higher diffusivity (MD, AD, and RD) in females than in males and higher NDI and ODI in males than in females, which cannot be accounted for by the head size differences. While there are some studies reporting lower MD values in males than in females in young adults and adolescents (e.g., Lebel and Beaulieu, 2011; Herting et al., 2012), such sex differences are often more regionally specific and not universally detected across studies (e.g., Lebel et al., 2008; Tamnes et al., 2010). Nonetheless, we did observe greater MD and AD over the entire WM skeleton in females than in males in our recent large-scale multi-cohort study (total *N* > 20,000) that covered most of the adult life span (Beaudet et al., 2020), suggesting that the greater diffusivity in females is not unique to this sample. With regard to NODDI, one study with a young to middle-age sample (age range of 18–55 years) reported a robustly higher NDI and ODI in males than in females (Kodiweera et al., 2016), similar to our findings; however, most studies do not report any sex differences in childhood and adolescence (Genc et al., 2017; Mah et al., 2017; Dimond et al., 2020; Lynch et al., 2020).

Despite the main effects of sex, we did not detect strong evidence for the sex differences in the age-related variations in our data. It is consistent with prior studies that report no or minimal interaction between sex and age after post-childhood in DTI (Hsu et al., 2010; Hasan et al., 2010; Tamnes et al., 2010; Inano et al., 2011; Lebel et al., 2012; Pohl et al., 2016) or NODDI (Cox et al., 2016; Kodiweera et al., 2016; Slater et al., 2019; Lynch et al., 2020). It suggests that any sex-related differences in the WM properties develop relatively early in development. Indeed, some studies reported steeper age-related changes in both FA and MD in boys than girls during childhood (Simmonds et al., 2014; Reynolds et al., 2019). However, further studies are needed to determine factors that may influence apparent sex differences in the WM properties and their rate of change with age in specific cohorts, such as body mass index, physical and intellectual activities, and other behavioral differences between the sexes that may modulate the WM properties.

### Potential Limitations

As we describe more in detail in Tsuchida et al. (2020), our sample from the MRi-Share database is drawn from students undergoing university-level education in Bordeaux, and as such, not necessarily a representative sample of healthy young adults. As a consequence, our sample is dominated by female participants, for example, and likely have different socio-demographic backgrounds and levels of education than the rest of the population of the same age range. They are also not guaranteed to be perfectly “healthy,” as the i-Share study, from which the MRi-Share participants were drawn, was designed to investigate the physical and mental health of students, and did not exclude those with a past or current history of mental illness, alcohol intake, smoking habits, and/or use of any recreational drugs and psychotropic medications. While this undoubtedly increases the variance unaccounted for in our data, it also makes our data more representative of the sampled population.

The MRi-Share database is also currently cross-sectional, limiting our inference of maturational trajectory from the age-related variations in the data. The analysis of age effects based on cross-sectional data has been shown to lead to spurious findings unsupported from longitudinal analysis, especially when using quadratic models to describe non-linear patterns of age-related changes (Fjell et al., 2010; Pfefferbaum and Sullivan, 2015). In our sample with a relatively limited age range of 18–26 years, we found that linear age trends were sufficient for characterizing age-related variations in the data, thus avoiding some of the pitfalls of fitting quadratic age models. While we still need to exert caution when interpreting the apparent age-related variations in our data, our findings were found to be broadly consistent with the known age-related trajectories in WM properties.

Though our DWI preprocessing pipeline included standard steps with susceptibility and eddy-current distortion correction and was similar to the official UKB DWI pipeline [with additional denoising using non-local means filter (Coupe et al., 2008, 2011)], our study did not make use of additional preprocessing steps such as bias field correction and Gibbs ringing correction. Recent work has highlighted the potential impact of such preprocessing choices on diffusion metrics and the observed age associations (Maximov et al., 2019). We also used the version of Eddy (patch 5.0.10) before the option to correct for within-volume movement (Andersson et al., 2017) and interactions between susceptibility and motion (Andersson et al., 2018) implemented in the latest version of the tool. Future investigations with this dataset may benefit from the updated preprocessing pipeline that incorporates these steps and examine the reliability of the findings from the current study.

Regarding the specific methodology for characterizing the regional WM properties, we used the ROIs based on the JHU ICBM-DTI-81 white matter labels atlas, computing the mean DTI/NODDI values within regions with high WM probability based on the multi-channel tissue segmentation with T1 and FLAIR scans. The ROIs in this atlas represent the WM regions with relatively well-organized structures that are clearly visible in the color-coded map of the tensor fields and should not be conflated with tracts obtained through tractography-based methods: The naming of these ROIs is based on the primary WM fiber population passing through the region, but these ROIs often represent a limited portion of a given tract, with arbitrary boundaries, and also may contain different fiber populations. For example, the corticospinal tract (CST) ROI in this atlas represents a portion of the CST at the level of medulla and pons, whereas the CST in the tractography-based methods usually refers to the fiber population that spans from corona radiata, passing through the internal capsule, then to the midbrain (Thiebaut de Schotten et al., 2011; Chenot et al., 2019). Another example is the sagittal stratum (SS) ROI, which, according to the authors of the atlas, includes both the inferior longitudinal fasciculus and the projection fibers from the internal capsule, therefore including both projection and association fibers (Mori et al., 2008). We also note that recent anatomical studies have seriously questioned the presence of superior fronto-occipital fasciculus (SFO) in humans (Türe et al., 1997; Forkel et al., 2014; Meola et al., 2015; Liu et al., 2020). Thus, this ROI most likely represents anterior thalamic radiation, as has been noted by the authors (Mori et al., 2008).

Although tractography-based methods allow a more direct characterization of any given tract in the WM, averaging of diffusion metrics along the entire length of tracts of interest can be problematic, in particular for DTI metrics, which can vary considerably along the tract due to the variability in the fiber tract geometry (Lebel et al., 2008; Vos et al., 2012). For this reason, more detailed comparisons of metrics at arbitrary points along the tract (“tract profiling”) have been proposed (Jones et al., 2005; Colby et al., 2012; Yeatman et al., 2012; Cousineau et al., 2017). Regardless of how to sample values from the tracts of interests, the choice of specific tracts to be extracted, the tracking or extraction criteria (seeding and exclusion regions for tracking specific tracts, or inclusion or exclusion criteria when extracting specific tracts from a whole-brain tractogram), tracking algorithms and their hyperparameters can complicate comparisons across studies (Côté et al., 2013). To avoid the bias introduced by study-specific protocols, a number of automated or semi-automated methods to extract major WM tracts have been proposed in recent years (Zhang et al., 2008; Yendiki et al., 2011; Yeatman et al., 2012; Wassermann et al., 2016; Wasserthal et al., 2018; Warrington et al., 2020), but no one method has been applied widely to characterize age-related changes in WM properties (Lebel et al., 2019). Also, more work is needed to assess the reproducibility and anatomical validity of different protocols for tract reconstructions (Rheault et al., 2020).

Within the studies using the ROI-based approach, and in particular the ROIs based on the same JHU ICBM-DTI-81 atlas, many use the framework of Tract-Based Spatial Statistics (TBSS, Smith et al., 2006), included with the FSL package. TBSS was developed to overcome the limitations of voxel-based analyses as applied to DTI metrics, namely the difficulty of aligning complex fiber architecture across subjects and the problem of smoothing images with highly heterogeneous noise such as FA. Its approach is to project the highest local FA values onto the non-linearly aligned group average or a template FA map that has been “skeletonized” by only taking the regions with maximal FA values with low inter-subject variability (Smith et al., 2006). The DTI or any other maps of diffusion metrics can then be projected to the FA-based skeleton to perform a voxel-based comparison within the skeleton or an ROI-based comparison using the atlas, such as the JHU atlas used in the present study. The focus on the WM skeleton with high FA values across subjects resolves the issue of alignment and correspondence across multiple subjects, but by design, it biases the characterization of the WM microstructural properties to the very small portion of WM inside the skeleton that is only one voxel in width, with relatively simple fiber orientations (Lebel et al., 2019). When used together with the ROI-based approach, the number of voxels contributing to the analyses are further reduced. In the present study, we used less restrictive sampling based on the WM probability map rather than the TBSS-style FA skeleton to allow for a more complete characterization of the regional microstructural properties. This approach also allowed for the direct comparison of the variations in the regional volume based on the Jacobian-modulated WM probability map and the variations in the microstructural properties in the same region. The inclusion of voxels outside the FA skeleton likely explain the relative lack of age or sex effects for mean FA values in our study, since it averages over regions with more complex fiber geometry and makes it difficult to dissociate changes related to the axonal diffusion properties from those related to the complexity of fiber orientations. However, multi-component tissue models such as NODDI can offer more specific inferences about the variations or differences in the microstructural properties without restricting the analysis to the WM skeleton, as we demonstrated in our study.

Another critical difference between the TBSS-based approach and the current study is the method of spatial normalization: after non-linear alignment of FA map to the template space, the TBSS projects the highest FA values onto a template FA skeleton in the standard space. Although it is meant to improve the alignment of the core of WM tracts, concerns have been raised with regard to the anatomical inaccuracies introduced by such a method (Bach et al., 2014). In the present study, we used the “Unified Segmentation” framework (Ashburner and Friston, 2005) to perform spatial normalization based on tissue segmentation of the structural scans, a common approach in voxel-based morphometry studies (e.g., Takao et al., 2011; Powell et al., 2012; Shiino et al., 2017). The non-linear deformation field obtained from the spatial normalization of the structural scans was then applied to DTI and NODDI maps, together with affine transformations that co-register these maps to the reference T1 scan of each subject. Although this is not necessarily the best available method to non-linearly align images (Klein et al., 2009), we believe that the sampling and averaging of values within the regions comprising hundreds of voxels (or thousands, in many ROIs), defined based on both the template atlas label and subject-specific WM probability map, would limit the effects of small misalignments, especially with the large sample size in our study. Having said that, the robustness of the findings should be confirmed using state-of-the-art methods to align images, such as registrations based on diffusion tensor images (Zhang et al., 2006) or fiber orientation distributions (Raffelt et al., 2011).

## Conclusion

In a large cohort of university students, we found a widespread increase in NDI, with a more regionally specific increase in ODI, indicating a continuing modulation of WM properties at this age range. We also demonstrated the distinct patterns of interrelations among the estimated age effects on different WM properties that were consistent with remodeling of myelin in post-adolescence. We did not find any evidence for a strong sex dependency in the patterns of age-related variations. These findings highlight the complexity of the patterns of regional WM properties and individual variations in such patterns. Although we focused on the basic characterization of age and sex effects in the present study, they represent a small portion of the variance in data, and there are large individual differences in the regional WM volumes and microstructure. Future studies should investigate how the maturational processes in the WM influence, or are influenced by, genetic, cognitive, behavioral, lifestyle and social factors, and how they are altered in neuropsychiatric conditions that manifest in early adulthood.

## Data Availability Statement

The datasets presented in this article are not readily available because to access i-Share and MRi-Share de-identified data, a request can be submitted to the i-Share Scientific Collaborations Coordinator (ilaria.montagni@u-bordeaux.fr) with a letter of intent (explaining the rationale and objectives of the research proposal), and a brief summary of the planned means and options for funding. The i-Share Steering Committee will assess this request, and provide a response (principle agreement, request to reformulate the application or for further information, refusal with reasons). If positive, applicants will have to complete and return an application package which will be reviewed by the principal investigator, the Steering Committee, and the operational staff. Reviews will be based on criteria such as the regulatory framework and adherence to regulations (access to data, confidentiality), the scientific and methodological quality of the project, the relevance of the project in relation to the overall consistency of the cohort in the long term, the complementarity/competition with projects planned or currently underway, ethical aspects. De-identified data (and data dictionaries) will be shared after (i) final approval of the application, and (ii) formalization of the specifics of the collaboration. The JHU WM phenotypes and associated QC metrics presented in this study are available in the Dryad repository (https://doi.org/10.5061/dryad.cvdncjt4m), and source codes for the statistical analysis presented are available on GitHub (https://github.com/atsuch/MRiShare_regionalWM_Age_analysis) or Zenodo (https://doi.org/10.5281/zenodo.5072215) repository associated with the Dryad repository. Requests to access the datasets should be directed to Ilaria Montagni, ilaria.montagni@u-bordeaux.fr.

## Ethics Statement

The studies involving human participants were reviewed and approved by the Comité de Protection des Personnes SUD-OUEST et Outre-Mer III. The patients/participants provided their written informed consent to participate in this study.

## Author Contributions

BM, CT, and SD contributed to conception and design of the study. AT and AL organized and processed imaging data to obtain JHU regional phenotypes described in the manuscript. AP and NB contributed to the QC of the image processing. AT and BM performed the statistical analysis and wrote the first draft of the manuscript. All authors contributed to manuscript revision, read, and approved the submitted version.

## Conflict of Interest

The authors declare that the research was conducted in the absence of any commercial or financial relationships that could be construed as a potential conflict of interest.

## Publisher’s Note

All claims expressed in this article are solely those of the authors and do not necessarily represent those of their affiliated organizations, or those of the publisher, the editors and the reviewers. Any product that may be evaluated in this article, or claim that may be made by its manufacturer, is not guaranteed or endorsed by the publisher.

## Footnotes

1
www.i-share.fr

2
https://www.fil.ion.ucl.ac.uk/spm/

3
http://surfer.nmr.mgh.harvard.edu/

4
https://fsl.fmrib.ox.ac.uk

5
https://dipy.org

6
https://scikit-learn.org/stable/index.html

7
https://scilpy.readthedocs.io/en/latest/
