# Supplementary material for: Age-Related Variations in Regional White Matter Volumetry and Microstructure During the Post-adolescence Period: A Cross-Sectional Study of a Cohort of 1,713 University Students
Source: Front Syst Neurosci. 2021 Aug 3;15:692152. doi: 10.3389/fnsys.2021.692152 (PMC8369154; doi:10.3389/fnsys.2021.692152)
Supplement: Supplementary file 1 [file Data_Sheet_1.pdf]

Supplementary Material

1. The effect of phenotypic and QC metric outlier removal and inclusion of a global QC metric in the model

While the quality of the acquired image and specific processing steps were visually reviewed and verified for all subjects for the structural scans (T1w and FLAIR images), we focused on identifying the outliers in a number of quantitative QC metrics for the DWI pipeline, then reviewing the subject-specific QC images for those subjects. Both the visual QC images and quantitative QC metrics were mainly related to the quality of DWI data: for example, visual QC images included the plots of relative motion (measured as the mean relative root mean square (RMS) of the displacement), the number of outlier slices as identified by Eddy tool (Bastiani et al., 2019) and percent outliers as determined by '3dTOutcount' tool from AFNI (Cox, 1996) per each frame, and contrast-to-noise ratio (CNR) and temporal signal-to-noise ratio (tSNR) images as well as midsagittal plots of raw images for each b-values, and maps of DTI residuals and "physically implausible voxels" where the mean intensity level of the b = 0 images is below one or more nonzero b-value images (Tournier et al., 2011). The quantifiable QC metrics represented their numerical summary. It included 1) the mean relative total RMS and "restricted" RMS, 2) the mean number of Eddy-based outlier slices and AFNI-based percent voxels per volume, 3) the mean CNR (for each b > 0 image) and tSNR (for each b-value) inside the brain, and 4) the mean DTI residuals and fraction of implausible voxel inside brain mask (for their distribution, see Supplemental Material in Tsuchida et al., (2020)). We additionally computed the image similarity between the spatially normalised individual image and the corresponding cohort average map (the WM tissue map, and DTI and NODDI maps) using Pearson's correlation to spot any subjects whose spatially normalised image deviated significantly from the cohort average.

Although the reviewing of individual QC images for subjects who had an outlier value in one or more quantitative DWI QC metrics revealed some indication of within-volume motion in those with multiple outlier QC values, the number of volumes affected was relatively small even in the worst case, and it was not immediately clear if the image quality rendered the data unusable. For this reason, we kept the entire sample for the present study but checked the effect of the quality metrics by 1) removing the subjects with an outlier value in any of the quantitative QC metrics or phenotypic values and 2) including a global metric of image quality as a covariate in the model.

For DTI and NODDI metrics, we used a total of 26 QC metrics (18 related to DWI quality and eight similarity metrics representing the quality of spatial normalisation). We used Tukey's method (Tukey, 1977) to identify "far out" outliers whose QC values were above three times the interquartile range (IQR) for any of the QC metrics (all the QC metrics were transformed if necessary so that the higher value indicated the lower quality). It identified 79 subjects (4.6% of the total sample) as having at least one outlier value in the QC metrics. For each DTI/NODDI metric in each JHU ROI, we used the same method to identify and remove the phenotypic outliers, removing up to 102 subjects, or about 6.0% of the total sample. We then applied the same model described in the main manuscript to check the impact of the outlier removal. In addition, to further control for any remaining effect of image quality, we used the mean relative RMS as a covariate in the model to check the robustness of our findings. The mean relative RMS is a measure of in-scanner motion, and a recent study has

demonstrated its impact on various DWI-derived metrics, including DTI and NODDI (Pines et al., 2020). Supplemental Figure 1 shows the visual comparison of age and sex effects with (“noOL”) or without (“main”) the outlier removal, and with outlier removal plus the additional inclusion of RMS in the model (“noOL + RMS”). Overall, the cleaning of data with outlier removal slightly improves the sensitivity to the age effects on diffusivity metrics and NDI. The impact on the estimated sex effects is minimal. The additional inclusion of RMS does not have any discernible effect on either age or sex effect estimates in our data.

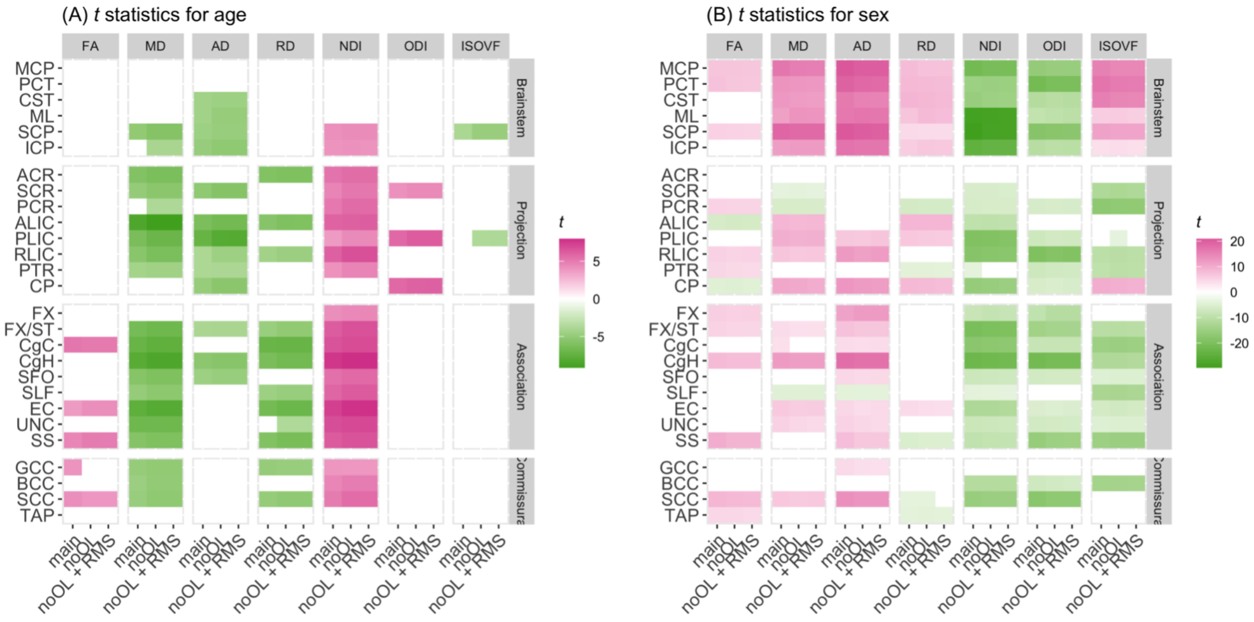

Supplemental Figure 1. The effect of outlier removal and addition of RMS in the model on DTI/NODDI phenotypes.
Effects of QC and phenotypic outlier removal and inclusion of RMS on the estimated age and sex effects in the regional DTI/NODDI phenotypes. The *t* statistics for (A) age and (B) sex effects are shown for each DTI/NODDI metric in the JHU ROIs. Each column represents *t* statistics derived from the analysis with no outlier removal (“main”, i.e. identical to the analysis described in the main text), with outlier removal (“noOL”), and with outlier removal plus the inclusion of RMS in the model (“noOL + RMS”). Those that did not survive Bonferroni corrections for multiple comparisons were filtered out (set to 0) to facilitate comparisons of significant results. See Table 1 of the main text for the full names of abbreviated ROIs.

Even though the quality of both tissue segmentation and spatial normalisation of segmented tissues were visually verified for all participants, we investigated the impact of similar QC and phenotypic outliers on the regional WM volume analyses as well. For this, we used the following quantifiable QC metrics related to SPM12-based tissue segmentation quality: 1) tissue-specific SNR computed for grey matter (GM), WM, and cerebrospinal fluid (CSF) for both T1w and FLAIR images, 2) GM-to-WM and WM-to-CSF CNR for T1w and FLAIR images, 3) FLAIR to T1 coregistration cost function, and 4) Freesurfer-based Euler number (Dale et al., 1999). The Euler number is a measure of cortical complexity computed during Freesurfer-based surface reconstruction, but it has been demonstrated to be a highly reliable measure of image quality (Rosen et al., 2018). We also used the image similarity of the individual WM tissue map to the cohort average WM map to quantify the

quality of the spatial normalisation. We identified the "far out" outliers across the total of 13 QC metrics, which identified only 18 QC outlier subjects, or about 1% of the sample. Similar to the comparison performed for DTI/NODDI metrics, we compared the effects of removing these QC outliers as well as individual phenotypic outliers removing up to 22 subjects (1.3% of the sample), and also the addition of Euler number as the global image QC metric. Not too surprisingly, the removal of such a small portion of the total sample did not have any discernible impact on the estimated age or sex effects. In our dataset, the addition of Euler number also did not have any measurable impact, likely due to the high overall quality of our structural images (the lowest Euler number that represents the poorest quality in our sample was -85; in comparison, a cutoff used to discard images for the dataset in Rosen et al., 2018 was -217).

2. The effect of global or regional volume corrections on the analyses

2.1. Effects of global volume correction on regional WM volumetry

The estimates of age and sex effects on the regional WM volumes partly depend on if and how global brain volume or head size is corrected. While we reported the age and sex effects on regional WM volumes without global volume correction in the main manuscript, such correction is particularly important when quantifying the sex differences since males typically have larger head size than females. We have also previously shown that the total WM volume (TWMV) significantly increased with age in this sample, only when controlling for overall head size by including TIV in the model (Tsuchida et al., 2020). Here, we investigated how the estimates of age- and sex-related variations in the regional WM volumes were modulated by the inclusion of TIV or TWMV in the model. We tested and compared the following variations of our primary models (1) and (2) described in the main manuscript:

1) "noVol": a model without any global volume correction (i.e. out primary model)

2) "TIV": a model that includes the TIV as a covariate

3) "TWMV": a model that includes the TWMV as a covariate

4) "TIV + TWMV": a model that includes both TIV and TWMV

Supplemental Figure 2 compares the total variance explained by each of the four models (adjusted R^2), and also shows their impact on the *t* statistics for age and sex. Not surprisingly, the inclusion of either TIV or TWMV significantly increased the total amount of variance explained. The inclusion of global volumes also affects the estimated age effects. For example, the cingulum in the cingulate gyrus (CgC) showed a significant age-related volumetric increase only if the global volume is taken into account by including either the TIV or TWMV. Other ROIs showed significant age-related increases when the TIV was controlled for, but not when TWMV was used instead, or vice versa (e.g. superior corona radiata (SCR), superior longitudinal fasciculus (SLF), and superior cerebellar peduncle (SCP)). Notably, the cingulum in the hippocampus (CgH) showed a significant age-related volumetric increase regardless of how or if the global volume was controlled.

For sex effects, males had significantly larger WM volumes than females across all the ROIs if the global volume was not taken into account. Many of these differences disappeared when TIV or TWMV was included in the model, and in a few cases, reversed, with females showing significantly larger relative volume than males, most notably in SCP. However, some ROIs exhibited attenuated

but still significant sex differences, with males having larger volumes than females, after global volume corrections (e.g. external capsule (EC), uncinate fasciculus (UNC)).

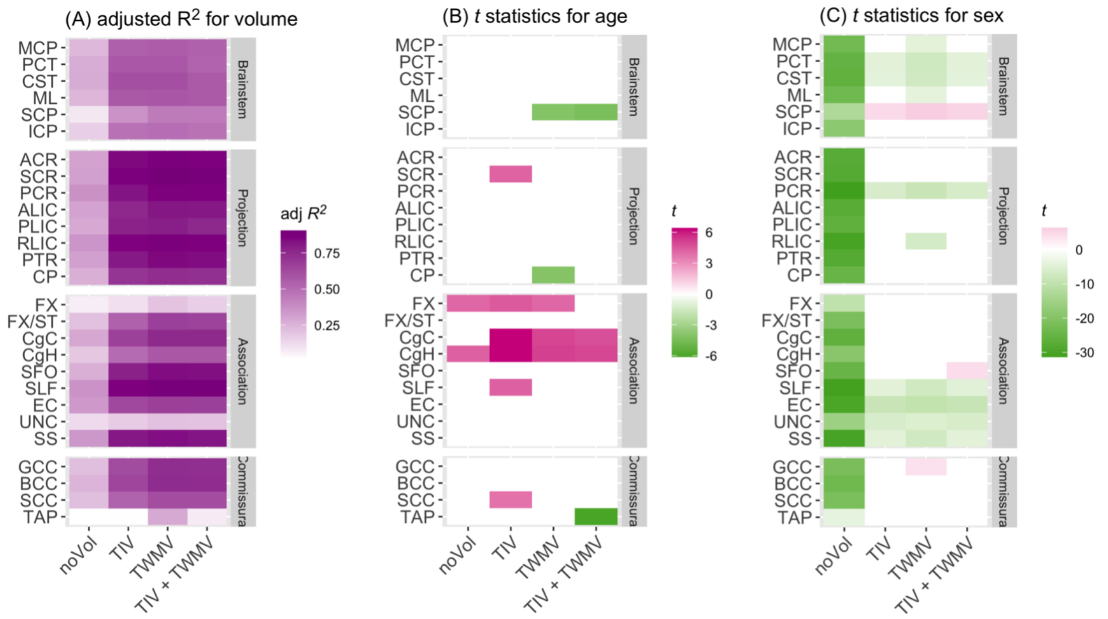

Supplemental Figure 2. Effects of global volume correction on the total variance explained and the age and sex effects in the WM JHU ROI volumes.
Comparisons of (A) adjusted R^2, (B) *t* statistics for age and (C) sex in the four models described in the text. Those that did not survive Bonferroni corrections for multiple comparisons were filtered out (set to 0) to facilitate comparisons within significant results. See Table 1 of the main text for the full names of abbreviated ROIs.

2.2. Effects of global or regional volume correction on DTI/NODDI metrics

Unlike for volumetry, the effects of global volume on mean DTI or NODDI values are not intuitive and less well-understood; however, we have previously shown that TIV significantly impacts mean DTI or NODDI metrics in WM skeleton or WM mask (Beaudet et al., 2020) (Beaudet et al., 2020; Tsuchida et al., 2020). For the regional mean values of DTI/NODDI metrics, a previous study has demonstrated that the mask volume for a given ROI could impact the mean DTI value inside the ROI, presumably due to the partial volume effect (Vos et al., 2011). Here we investigated the impact of the global volume (TIV) or the local ROI volume (ROIV) on the mean DTI/NODDI metrics and the estimates of age and sex effects. We tested and compared the following models:

1) “noVol”: a model without any global or local volume correction (i.e. our primary model)

2) “TIV”: a model that includes the TIV

3) “ROIV”: a model that includes the ROIV

4) “TIV + ROIV”: a model that includes both the TIV and ROIV

Supplemental Figure 3 compares the changes in model quality across the four models. The Bayesian information criterion (BIC) values indicated that the most parsimonious model (those with the lowest BIC value) varied across ROIs and DTI/NODDI metrics. Having global or local volumes as covariates tended to improve the model fit across metrics in a number of ROIs. The overall variance explained by the inclusion of these volumes increased slightly in many ROIs, particularly for NDI in which inclusion of TIV visibly increased adjusted R^2.

Supplemental Figure 4 summarises the contributions of TIV and ROIV on DTI/NODDI metrics directly by visualising the *t* statistics of TIV and ROIV terms in the models (2) and (3), respectively. It also shows their impact on the estimated age and sex effects across the models. Larger TIV or ROIV was associated with higher NDI in many ROIs, although NDI in a few ROIs were negatively associated with TIV (anterior corona radiata (ACR), TAP). They were also associated with higher IsoVF in most ROIs as well, except in the brainstem ROIs. For DTI metrics, the effects of TIV and ROIV were more variable and mixed across different ROIs.

Despite the significant relationships between global and local volumes with the mean DTI/NODDI values in these ROIs, their impact on age effect estimates was minimal. Most of the age-related variations observed for DTI/NODDI metrics in the 27 ROIs remained significant across the models with or without TIV or ROIV as covariates. The sex differences were modulated slightly by the inclusion of TIV across many metrics and ROIs, typically attenuating the degree of significance.

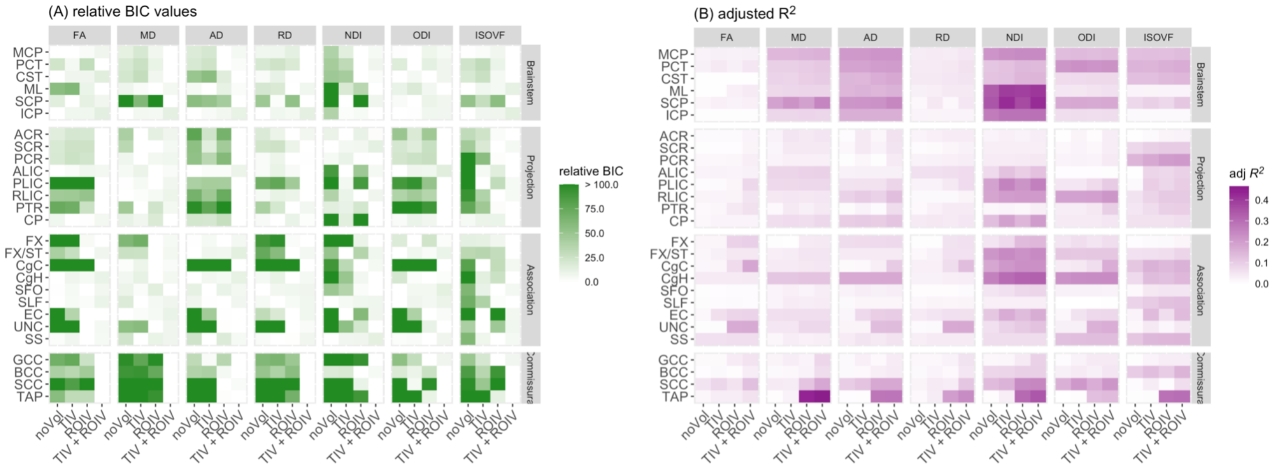

Supplemental Figure 3. Comparisons of model quality for models with or without global and/or local volume correction for the mean DTI/NODDI values in JHU ROIs.
Comparisons of (A) relative BIC values and (B) adjusted R^2 in the four models described in the text. See Table 1 of the main text for the full names of abbreviated ROIs.

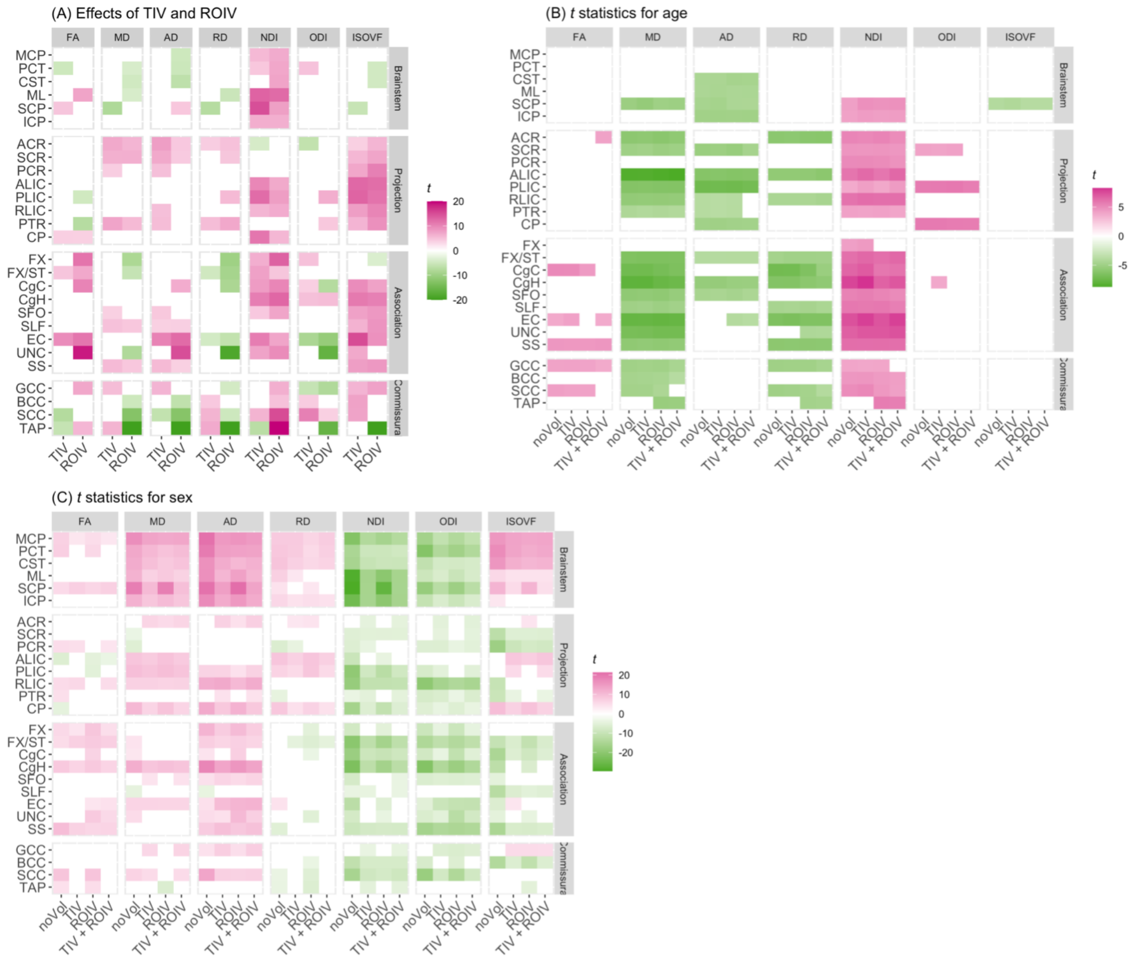

Supplemental Figure 4. Effects of global and local volumes on the mean DTI/NODDI values in JHU ROIs and their impact on the estimated age and sex effects.
Comparisons of the *t* statisics for (A) TIV and ROIV in "TIV" and "ROIV" models, respectively, (B) age and (C) sex effects across the four models described in the text. Those that did not survive Bonferroni corrections for multiple comparisons were filtered out (set to 0) to facilitate comparisons within significant results. See Table 1 of the main text for the full names of abbreviated ROIs.

3. Complete tables of model results and age scatter plots for each metric and ROI

Due to limited space, we provided a table of raw parameter estimates only for the age effect in the main manuscript. Here we summarise parameter estimates (β), p values, and generalised η^2 for both age and sex effects, as well as for the interaction between age and sex (although none of the interactions survived the correction for multiple comparisons), and also provide adjusted R^2 for each metric and ROI. We also provide the raw scatter plots of age effects for each metric and ROI.

Supplemental Table 1. Model results for WM JHU ROI volumes.
The raw parameter estimate (β) values and their 95 % confidence intervals (in square brackets) are in mm^3/year for Age and Age x Sex, and in mm^3 for Sex effects. The first columns give the abbreviated JHU ROIs (see Table 1 of the main text for their full names). Statistical significance symbols

(uncorrected for multiple comparisons) *: 0.05 < p <0.001, **: 0.001 < p < 0.0001, ***: p < 0.0001. Bold symbols indicate Bonferroni-corrected significant p-values.

	Age			Sex			Age X Sex			
	β [95%CI]	*p value*	η^2_G	*β [95%CI]*	*p value*	η^2_G	*β [95%CI]*	*p value*	η^2_G	*adj.* R^2
Brainstem										
MCP	-25.2 [-56.6, 6.3]	>0.1	0.001	**-664.7*** [-721.4, -607.9]**	**<0.00001**	**0.236**	16.6 [-14.9, 48.1]	>0.1	<.001	0.235
PCT	-2.7 [-6.1, 0.6]	>0.1	0.001	**-78.5*** [-84.6, -72.4]**	**<0.00001**	**0.273**	2.4 [-1.0, 5.7]	>0.1	<.001	0.272
CST	-1.8 [-4.6, 1.1]	>0.1	<.001	**-67.9*** [-73.1, -62.8]**	**<0.00001**	**0.281**	1.6 [-1.3, 4.4]	>0.1	<.001	0.28
ML	-1.2 [-2.6, 0.2]	0.0986	0.001	**-30.4*** [-32.9, -27.8]**	**<0.00001**	**0.242**	1.0 [-0.4, 2.5]	>0.1	<.001	0.241
SCP	-2.3* [-4.0, -0.6]	0.0076	0.004	**-19.6*** [-22.7, -16.6]**	**<0.00001**	**0.085**	-0.1 [-1.8, 1.6]	>0.1	<.001	0.087
ICP	-0.4 [-2.0, 1.3]	>0.1	<.001	**-27.7*** [-30.6, -24.7]**	**<0.00001**	**0.167**	0.6 [-1.0, 2.2]	>0.1	<.001	0.166
Projection										
ACR	3.2 [-10.4, 16.9]	>0.1	<.001	**-345.1*** [-369.7, -320.5]**	**<0.00001**	**0.307**	4.1 [-9.6, 17.8]	>0.1	<.001	0.307
SCR	5.6 [-7.8, 19.1]	>0.1	<.001	**-345.5*** [-369.7, -321.3]**	**<0.00001**	**0.314**	-0.0 [-13.5, 13.4]	>0.1	<.001	0.315
PCR	3.3 [-3.5, 10.1]	>0.1	<.001	**-194.8*** [-206.9, -182.6]**	**<0.00001**	**0.365**	-0.7 [-7.4, 6.1]	>0.1	<.001	0.366
ALIC	1.7 [-3.1, 6.5]	>0.1	<.001	**-120.0*** [-128.6, -111.4]**	**<0.00001**	**0.304**	-0.7 [-5.5, 4.1]	>0.1	<.001	0.304
PLIC	-0.5 [-7.3, 6.4]	>0.1	<.001	**-164.3*** [-176.7, -152.0]**	**<0.00001**	**0.286**	0.6 [-6.3, 7.4]	>0.1	<.001	0.285
RLIC	-0.9 [-5.1, 3.2]	>0.1	<.001	**-114.4*** [-121.8, -107.0]**	**<0.00001**	**0.349**	0.1 [-4.0, 4.2]	>0.1	<.001	0.349
PTR	-1.4 [-8.5, 5.7]	>0.1	<.001	**-182.5*** [-195.2, -169.7]**	**<0.00001**	**0.315**	0.4 [-6.7, 7.5]	>0.1	<.001	0.314
CP	-3.9 [-8.1, 0.2]	0.0636	0.001	**-93.9*** [-101.4, -86.4]**	**<0.00001**	**0.262**	1.3 [-2.9, 5.4]	>0.1	<.001	0.261
Association										
FX	**2.6*** [1.3, 3.8]**	**<0.00001**	**0.009**	**-11.6*** [-13.8, -9.4]**	**<0.00001**	**0.057**	-0.0 [-1.3, 1.2]	>0.1	<.001	0.068
FX/ST	-0.2 [-1.9, 1.5]	>0.1	<.001	**-33.6*** [-36.7, -30.5]**	**<0.00001**	**0.209**	-0.6 [-2.3, 1.1]	>0.1	<.001	0.209
CgC	8.3** [3.8, 12.8]	0.0003	0.005	**-107.9*** [-116.0, -99.8]**	**<0.00001**	**0.283**	-1.5 [-6.0, 3.0]	>0.1	<.001	0.291

CgH	**5.1*** [2.7, 7.4]**	**<0.00001**	**0.009**	**-41.0*** [-45.2, -36.8]**	**<0.00001**	**0.173**	-1.0 [-3.4, 1.3]	>0.1	<.001	0.185
SFO	0.5 [-0.5, 1.4]	>0.1	<.001	**-21.6*** [-23.4, -19.9]**	**<0.00001**	**0.262**	0.1 [-0.8, 1.1]	>0.1	<.001	0.263
SLF	6.3 [-5.6, 18.3]	>0.1	<.001	**-339.3*** [-360.8, -317.9]**	**<0.00001**	**0.36**	-1.9 [-13.8, 10.1]	>0.1	<.001	0.361
EC	3.8 [-2.6, 10.2]	>0.1	<.001	**-174.4*** [-186.0, -162.9]**	**<0.00001**	**0.339**	4.4 [-2.1, 10.8]	>0.1	<.001	0.341
UNC	-0.3 [-1.3, 0.7]	>0.1	<.001	**-14.0*** [-15.7, -12.2]**	**<0.00001**	**0.125**	0.1 [-0.9, 1.1]	>0.1	<.001	0.124
SS	-0.8 [-4.7, 3.1]	>0.1	<.001	**-107.4*** [-114.3, -100.4]**	**<0.00001**	**0.348**	-0.2 [-4.0, 3.7]	>0.1	<.001	0.348
Commissural										
GCC	5.1 [-12.8, 22.9]	>0.1	<.001	**-354.4*** [-386.5, -322.2]**	**<0.00001**	**0.215**	0.4 [-17.5, 18.2]	>0.1	<.001	0.215
BCC	21.7 [-4.5, 47.9]	>0.1	0.001	**-563.9*** [-611.1, -516.6]**	**<0.00001**	**0.243**	-15.0 [-41.2, 11.2]	>0.1	<.001	0.245
SCC	31.5* [3.2, 59.7]	0.0289	0.002	**-554.7*** [-605.5, -503.8]**	**<0.00001**	**0.211**	3.0 [-25.2, 31.2]	>0.1	<.001	0.214
TAP	-2.7* [-4.5, -0.9]	0.0039	0.005	**-6.5** [-9.7, -3.2]**	**0.0001**	**0.009**	-0.2 [-2.0, 1.6]	>0.1	<.001	0.013

Supplemental Table 2. Model results for mean FA in JHU ROIs.
The raw parameter estimate (β) values and their 95 % confidence intervals (in square brackets) are x10^{-3}/year change in FA for Age and Age x Sex effects and x10^{-3} for Sex effect. The first columns give the abbreviated JHU ROIs (see Table 1 of the main text for their full names). Statistical significance symbols (uncorrected for multiple comparisons) *: 0.05 < p <0.001, **: 0.001 < p < 0.0001, ***: p < 0.0001. Bold symbols indicate Bonferroni-corrected significant p-values.

	Age			Sex			Age X Sex			
	β [95%CI]	*p value*	*η^2_G*	*β [95%CI]*	*p value*	*η^2_G*	*β [95%CI]*	*p value*	*η^2_G*	*adj. R^2*
Brainstem										
MCP	-0.0 [-0.6, 0.6]	>0.1	<.001	**4.0*** [2.9, 5.1]**	**<0.00001**	**0.027**	-0.2 [-0.8, 0.4]	>0.1	<.001	0.026
PCT	-0.7 [-1.7, 0.4]	>0.1	<.001	**7.1*** [5.1, 9.0]**	**<0.00001**	**0.029**	-0.0 [-1.1, 1.0]	>0.1	<.001	0.029
CST	-1.3* [-2.4, -0.1]	0.0312	0.003	0.4 [-1.6, 2.5]	>0.1	<.001	0.7 [-0.4, 1.9]	>0.1	<.001	0.001
ML	-1.1 [-2.4, 0.2]	>0.1	0.001	-0.8 [-3.2, 1.6]	>0.1	<.001	0.4 [-0.9, 1.8]	>0.1	<.001	−0.000

SCP	-0.3<br>[-1.1, 0.6]	>0.1	<.001	**4.5*****<br>**[2.9, 6.1]**	**<0.00001**	**0.017**	1.0*<br>[0.1, 1.9]	0.0238	0.003	0.019
ICP	-0.3<br>[-1.2, 0.7]	>0.1	<.001	2.2*<br>[0.5, 3.8]	0.0092	0.004	0.1<br>[-0.8, 1.0]	>0.1	<.001	0.003
Projection										
ACR	1.4**<br>[0.6, 2.2]	0.0003	0.008	-0.4<br>[-1.7, 1.0]	>0.1	<.001	0.0<br>[-0.7, 0.8]	>0.1	<.001	0.008
SCR	-0.6<br>[-1.4, 0.1]	0.0905	0.002	1.6*<br>[0.2, 2.9]	0.022	0.003	0.0<br>[-0.7, 0.8]	>0.1	<.001	0.004
PCR	0.6<br>[-0.3, 1.4]	>0.1	<.001	**4.1*****<br>**[2.5, 5.6]**	**<0.00001**	**0.016**	0.2<br>[-0.6, 1.0]	>0.1	<.001	0.015
ALIC	0.6<br>[-0.1, 1.2]	0.0966	0.002	**-3.7*****<br>**[-4.9, -2.6]**	**<0.00001**	**0.022**	0.1<br>[-0.5, 0.8]	>0.1	<.001	0.024
PLIC	-0.7*<br>[-1.4, -0.1]	0.0185	0.003	-1.8*<br>[-2.9, -0.7]	0.0017	0.006	0.3<br>[-0.4, 0.9]	>0.1	<.001	0.007
RLIC	0.8*<br>[0.2, 1.5]	0.0111	0.004	**3.2*****<br>**[2.0, 4.3]**	**<0.00001**	**0.017**	0.3<br>[-0.4, 0.9]	>0.1	<.001	0.021
PTR	0.3<br>[-0.4, 1.1]	>0.1	<.001	**3.4*****<br>**[2.0, 4.7]**	**<0.00001**	**0.014**	-0.2<br>[-1.0, 0.5]	>0.1	<.001	0.012
CP	-1.2*<br>[-2.0, -0.4]	0.0039	0.005	**-3.5*****<br>**[-5.0, -2.0]**	**<0.00001**	**0.012**	0.7<br>[-0.1, 1.5]	0.0846	0.002	0.015
Association										
FX	0.7<br>[-0.3, 1.7]	>0.1	<.001	**5.4*****<br>**[3.6, 7.2]**	**<0.00001**	**0.019**	0.3<br>[-0.7, 1.4]	>0.1	<.001	0.019
FX/ST	0.9*<br>[0.1, 1.7]	0.0348	0.003	**4.0*****<br>**[2.5, 5.5]**	**<0.00001**	**0.016**	0.2<br>[-0.6, 1.0]	>0.1	<.001	0.018
CgC	**2.3*****<br>**[1.4, 3.1]**	**<0.00001**	**0.015**	1.2<br>[-0.4, 2.7]	>0.1	0.001	-0.2<br>[-1.1, 0.6]	>0.1	<.001	0.016
CgH	1.2*<br>[0.3, 2.2]	0.0093	0.004	**6.9*****<br>**[5.3, 8.6]**	**<0.00001**	**0.037**	0.3<br>[-0.6, 1.3]	>0.1	<.001	0.041
SFO	0.1<br>[-1.0, 1.2]	>0.1	<.001	2.6*<br>[0.6, 4.6]	0.0125	0.004	0.6<br>[-0.5, 1.7]	>0.1	<.001	0.003
SLF	1.0*<br>[0.3, 1.7]	0.0051	0.005	0.9<br>[-0.4, 2.2]	>0.1	0.001	0.2<br>[-0.5, 0.9]	>0.1	<.001	0.006
EC	**1.2****<br>**[0.6, 1.8]**	**0.0002**	**0.008**	-1.9*<br>[-3.0, -0.7]	0.0012	0.006	0.1<br>[-0.5, 0.7]	>0.1	<.001	0.016
UNC	1.0<br>[-0.1, 2.0]	0.0633	0.002	1.2<br>[-0.7, 3.1]	>0.1	<.001	0.1<br>[-0.9, 1.2]	>0.1	<.001	0.002
SS	**1.6*****<br>**[0.9, 2.3]**	**<0.00001**	**0.011**	**6.4*****<br>**[5.1, 7.6]**	**<0.00001**	**0.054**	-0.3<br>[-1.0, 0.4]	>0.1	<.001	0.062
Commissural										

GCC	**1.7*** [0.9, 2.5]**	**<0.00001**	**0.01**	-0.2 [-1.6, 1.3]	>0.1	<.001	-0.3 [-1.1, 0.5]	>0.1	<.001	0.009
BCC	0.7 [-0.0, 1.4]	0.0584	0.002	1.7* [0.5, 3.0]	0.0072	0.004	-0.5 [-1.2, 0.2]	>0.1	0.001	0.004
SCC	**1.4*** [0.7, 2.0]**	**<0.00001**	**0.01**	**5.1*** [4.0, 6.3]**	**<0.00001**	**0.043**	0.1 [-0.5, 0.8]	>0.1	<.001	0.053
TAP	2.0* [0.6, 3.3]	0.0036	0.005	**5.6*** [3.2, 8.0]**	**<0.00001**	**0.012**	0.4 [-0.9, 1.7]	>0.1	<.001	0.017

Supplemental Table 3. Model results for mean MD in JHU ROIs.
The raw parameter estimate (*β*) values and their 95 % confidence intervals (in square brackets) are in $x10^{-6}$ mm^2/sec/year for Age and Age x Sex, and in mm^2/sec for Sex effects. The first columns give the abbreviated JHU ROIs (see Table 1 of the main text for their full names). Statistical significance symbols (uncorrected for multiple comparisons) *: $0.05 < p < 0.001$, **: $0.001 < p < 0.0001$, ***: $p < 0.0001$. Bold symbols indicate Bonferroni-corrected significant *p*-values.

	Age			Sex			Age X Sex			
	β [95%CI]	*p value*	*η^2_G*	*β [95%CI]*	*p value*	*η^2_G*	*β [95%CI]*	*p value*	*η^2_G*	*adj. R2*
Brainstem										
MCP	-0.9* [-1.5, -0.2]	0.0144	0.003	**10.6*** [9.4, 11.8]**	**<0.00001**	**0.141**	0.5 [-0.2, 1.2]	>0.1	0.001	0.146
PCT	-1.9 [-3.8, 0.1]	0.0638	0.002	**23.8*** [20.2, 27.3]**	**<0.00001**	**0.091**	1.7 [-0.3, 3.6]	0.0972	0.001	0.094
CST	-2.6* [-4.7, -0.5]	0.0151	0.003	**24.0*** [20.2, 27.8]**	**<0.00001**	**0.083**	1.2 [-0.9, 3.3]	>0.1	<.001	0.086
ML	-2.4* [-4.4, -0.5]	0.0142	0.003	**20.6*** [17.1, 24.1]**	**<0.00001**	**0.072**	1.9 [-0.1, 3.8]	0.0584	0.002	0.076
SCP	**-2.0*** [-2.8, -1.2]**	**<0.00001**	**0.013**	**13.3*** [12.0, 14.7]**	**<0.00001**	**0.174**	0.8 [-0.0, 1.5]	0.0521	0.002	0.191
ICP	-2.1** [-3.2, -1.0]	0.0003	0.007	**12.2*** [10.2, 14.3]**	**<0.00001**	**0.075**	0.6 [-0.5, 1.7]	>0.1	<.001	0.083
Projection										
ACR	**-2.0*** [-2.7, -1.3]**	**<0.00001**	**0.02**	1.6* [0.4, 2.8]	0.0101	0.004	0.4 [-0.3, 1.0]	>0.1	<.001	0.024
SCR	**-1.3*** [-1.7, -0.8]**	**<0.00001**	**0.014**	**-1.7** [-2.6, -0.8]**	**0.0002**	**0.008**	0.4 [-0.1, 0.9]	>0.1	0.001	0.02
PCR	-1.3** [-2.0, -0.6]	0.0005	0.007	**-3.9*** [-5.2, -2.6]**	**<0.00001**	**0.02**	0.6 [-0.1, 1.4]	0.0793	0.002	0.024
ALIC	**-2.0*** [-2.5, -1.5]**	**<0.00001**	**0.039**	**3.7*** [2.8, 4.5]**	**<0.00001**	**0.041**	0.3 [-0.1, 0.8]	>0.1	0.001	0.085

PLIC	-1.4*** [-1.9, -1.0]	<0.00001	0.022	4.0*** [3.2, 4.8]	<0.00001	0.051	0.5* [0.1, 1.0]	0.0224	0.003	0.075
RLIC	-1.6*** [-2.2, -1.0]	<0.00001	0.018	3.5*** [2.5, 4.5]	<0.00001	0.026	0.6* [0.0, 1.1]	0.0491	0.002	0.045
PTR	-1.4*** [-2.1, -0.8]	<0.00001	0.01	-1.7* [-2.9, -0.5]	0.0051	0.005	0.7* [0.0, 1.3]	0.0451	0.002	0.012
CP	-2.2* [-4.1, -0.3]	0.0206	0.003	18.4*** [15.0, 21.7]	<0.00001	0.063	0.2 [-1.7, 2.0]	>0.1	<.001	0.066
Association										
FX	0.3 [-1.3, 1.9]	>0.1	<.001	3.6* [0.8, 6.5]	0.0123	0.004	0.2 [-1.4, 1.8]	>0.1	<.001	0.002
FX/ST	-2.2*** [-2.8, -1.5]	<0.00001	0.024	2.5*** [1.3, 3.7]	<0.00001	0.01	0.7* [0.1, 1.4]	0.0331	0.003	0.034
CgC	-2.0*** [-2.5, -1.4]	<0.00001	0.029	1.9** [0.9, 2.9]	0.0001	0.008	0.2 [-0.3, 0.8]	>0.1	<.001	0.041
CgH	-3.0*** [-3.8, -2.3]	<0.00001	0.033	8.3*** [7.0, 9.7]	<0.00001	0.076	0.8* [0.0, 1.5]	0.0439	0.002	0.114
SFO	-1.8*** [-2.4, -1.2]	<0.00001	0.019	1.3* [0.2, 2.4]	0.0178	0.003	0.3 [-0.3, 0.9]	>0.1	<.001	0.023
SLF	-1.3*** [-1.8, -0.8]	<0.00001	0.014	-2.1*** [-3.0, -1.2]	<0.00001	0.012	0.3 [-0.2, 0.8]	>0.1	<.001	0.023
EC	-1.7*** [-2.2, -1.3]	<0.00001	0.033	2.6*** [1.8, 3.4]	<0.00001	0.023	-0.0 [-0.4, 0.4]	>0.1	<.001	0.064
UNC	-1.8*** [-2.3, -1.3]	<0.00001	0.026	2.6*** [1.7, 3.5]	<0.00001	0.016	0.1 [-0.4, 0.6]	>0.1	<.001	0.047
SS	-2.0*** [-2.6, -1.3]	<0.00001	0.019	-0.1 [-1.3, 1.1]	>0.1	<.001	0.8* [0.1, 1.4]	0.0265	0.003	0.017
Commissural										
GCC	-1.7*** [-2.3, -1.0]	<0.00001	0.014	2.1** [0.9, 3.3]	0.0006	0.007	0.5 [-0.2, 1.1]	>0.1	0.001	0.021
BCC	-1.3*** [-1.9, -0.8]	<0.00001	0.012	-0.6 [-1.6, 0.4]	>0.1	<.001	0.7* [0.2, 1.3]	0.0103	0.004	0.012
SCC	-1.5*** [-2.1, -0.9]	<0.00001	0.013	3.6*** [2.5, 4.7]	<0.00001	0.024	0.9* [0.3, 1.5]	0.0051	0.004	0.038
TAP	-1.4* [-2.7, -0.1]	0.033	0.003	-2.9* [-5.2, -0.5]	0.0172	0.003	1.5* [0.2, 2.8]	0.0286	0.003	0.005

Supplemental Table 4. Model results for mean AD in JHU ROIs.

The raw parameter estimate (β) values and their 95 % confidence intervals (in square brackets) are in x10^{-6} mm^2/sec/year for Age and Age x Sex, and in mm^2/sec for Sex effects. The first columns give the abbreviated JHU ROIs (see Table 1 of the main text for their full names). Statistical significance

symbols (uncorrected for multiple comparisons) *: 0.05 < p <0.001, **: 0.001 < p < 0.0001, ***: p < 0.0001. Bold symbols indicate Bonferroni-corrected significant p-values.

	Age			Sex			Age X Sex			
	β [95%CI]	*p value*	η^2_G	*β [95%CI]*	*p value*	η^2_G	*β [95%CI]*	*p value*	η^2_G	*adj. R2*
Brainstem										
MCP	-1.4* [-2.6, -0.3]	0.0135	0.003	**21.7*** [19.7, 23.8]**	**<0.00001**	**0.203**	0.6 [-0.5, 1.8]	>0.1	<.001	0.208
PCT	-3.3* [-5.6, -1.0]	0.0046	0.004	**40.8*** [36.7, 44.9]**	**<0.00001**	**0.181**	2.2 [-0.1, 4.5]	0.0606	0.002	0.188
CST	**-5.6*** [-8.1, -3.1]**	**<0.00001**	**0.01**	**37.6*** [33.1, 42.0]**	**<0.00001**	**0.135**	2.7* [0.2, 5.2]	0.0336	0.002	0.147
ML	**-5.1*** [-7.4, -2.8]**	**<0.00001**	**0.009**	**34.8*** [30.6, 38.9]**	**<0.00001**	**0.136**	3.2* [0.9, 5.5]	0.0065	0.004	0.149
SCP	**-4.1*** [-5.9, -2.4]**	**<0.00001**	**0.01**	**33.4*** [30.2, 36.5]**	**<0.00001**	**0.199**	2.8* [1.1, 4.6]	0.0014	0.005	0.214
ICP	**-3.6*** [-5.0, -2.1]**	**<0.00001**	**0.012**	**22.7*** [20.2, 25.3]**	**<0.00001**	**0.148**	1.2 [-0.2, 2.6]	>0.1	0.001	0.163
Projection										
ACR	-1.4* [-2.4, -0.4]	0.0058	0.004	2.4* [0.6, 4.2]	0.0083	0.004	0.5 [-0.5, 1.5]	>0.1	<.001	0.007
SCR	**-2.6*** [-3.5, -1.6]**	**<0.00001**	**0.016**	-1.5 [-3.2, 0.2]	0.0921	0.002	0.6 [-0.4, 1.6]	>0.1	<.001	0.016
PCR	-1.3* [-2.5, -0.2]	0.0255	0.003	-1.6 [-3.7, 0.5]	>0.1	0.001	1.2* [0.0, 2.4]	0.0454	0.002	0.003
ALIC	**-2.8*** [-3.7, -2.0]**	**<0.00001**	**0.022**	2.4* [0.7, 4.0]	0.0041	0.005	0.7 [-0.2, 1.6]	>0.1	0.001	0.027
PLIC	**-3.4*** [-4.3, -2.5]**	**<0.00001**	**0.029**	**5.6*** [3.9, 7.2]**	**<0.00001**	**0.025**	1.3* [0.4, 2.2]	0.0048	0.004	0.055
RLIC	**-1.8** [-2.8, -0.9]**	**0.0001**	**0.008**	**10.5*** [8.8, 12.2]**	**<0.00001**	**0.08**	1.4* [0.4, 2.3]	0.0042	0.004	0.091
PTR	**-2.2** [-3.3, -1.1]**	**0.0001**	**0.009**	1.5 [-0.4, 3.5]	>0.1	0.001	0.9 [-0.2, 2.0]	>0.1	0.001	0.009
CP	**-5.8*** [-8.1, -3.5]**	**<0.00001**	**0.013**	**27.3*** [23.1, 31.5]**	**<0.00001**	**0.086**	1.7 [-0.6, 4.0]	>0.1	0.001	0.101
Association										
FX	1.1 [-0.8, 3.0]	>0.1	<.001	**19.7*** [16.2, 23.1]**	**<0.00001**	**0.069**	1.4 [-0.5, 3.3]	>0.1	0.001	0.071
FX/ST	**-3.0*** [-4.5, -1.5]**	**0.0001**	**0.009**	**10.2*** [7.5, 12.9]**	**<0.00001**	**0.031**	1.6* [0.1, 3.1]	0.04	0.002	0.04
CgC	-0.5	>0.1	<.001	**4.4***	**<0.00001**	**0.01**	0.1	>0.1	<.001	0.009

	[-1.6, 0.6]			**[2.3, 6.4]**			[-1.0, 1.3]			
CgH	**-3.6***<br>[-4.9, -2.3]**	**<0.00001**	**0.014**	**21.4***<br>[19.1, 23.7]**	**<0.00001**	**0.157**	1.7*<br>[0.4, 2.9]	0.0123	0.003	0.176
SFO	**-2.8***<br>[-4.0, -1.6]**	**<0.00001**	**0.012**	**5.2***<br>[3.0, 7.3]**	**<0.00001**	**0.013**	1.0<br>[-0.2, 2.2]	0.0947	0.002	0.025
SLF	-0.8<br>[-1.5, 0.0]	0.0578	0.002	**-3.0***<br>[-4.4, -1.6]**	**<0.00001**	**0.01**	0.7<br>[-0.1, 1.4]	>0.1	0.002	0.011
EC	-1.4**<br>[-2.2, -0.6]	0.0005	0.007	**3.3***<br>[1.9, 4.7]**	**<0.00001**	**0.012**	0.0<br>[-0.7, 0.8]	>0.1	<.001	0.02
UNC	-2.1*<br>[-3.5, -0.8]	0.0013	0.006	**6.3***<br>[3.9, 8.6]**	**<0.00001**	**0.016**	0.5<br>[-0.8, 1.8]	>0.1	<.001	0.021
SS	-1.2*<br>[-2.3, -0.1]	0.0279	0.003	**7.6***<br>[5.7, 9.5]**	**<0.00001**	**0.034**	0.9<br>[-0.2, 2.0]	0.099	0.002	0.037
Commissural										
GCC	-0.8<br>[-2.0, 0.4]	>0.1	<.001	**4.9***<br>[2.8, 7.1]**	**<0.00001**	**0.012**	0.5<br>[-0.7, 1.7]	>0.1	<.001	0.011
BCC	-1.5*<br>[-2.5, -0.5]	0.0027	0.005	2.9*<br>[1.2, 4.7]	0.0013	0.006	0.8<br>[-0.2, 1.8]	>0.1	0.001	0.01
SCC	-1.0<br>[-2.2, 0.3]	>0.1	0.001	**15.7***<br>[13.4, 18.0]**	**<0.00001**	**0.096**	2.0*<br>[0.7, 3.3]	0.0019	0.005	0.102
TAP	0.5<br>[-1.9, 2.8]	>0.1	<.001	2.6<br>[-1.6, 6.9]	>0.1	<.001	3.1*<br>[0.8, 5.5]	0.0095	0.004	0.005

Supplemental Table 5. Model results for mean RD in JHU ROIs.
The raw parameter estimate (*β*) values and their 95 % confidence intervals (in square brackets) are in x10^{-6} mm^2/sec/year for Age and Age x Sex, and in mm^2/sec for Sex effects. The first columns give the abbreviated JHU ROIs (see Table 1 of the main text for their full names). Statistical significance symbols (uncorrected for multiple comparisons) *: 0.05 < *p* <0.001, **: 0.001 < *p* < 0.0001, ***: *p* < 0.0001. Bold symbols indicate Bonferroni-corrected significant *p*-values.

	Age			Sex			Age X Sex			
	β [95%CI]	*p value*	η^2_G	*β [95%CI]*	*p value*	η^2_G	*β [95%CI]*	*p value*	η^2_G	*adj. R2*
Brainstem										
MCP	-0.6<br>[-1.3, 0.1]	0.0999	0.002	**5.0***<br>[3.8, 6.3]**	**<0.0000<br>1**	**0.036**	0.5<br>[-0.2, 1.2]	>0.1	0.001	0.038
PCT	-1.2<br>[-3.1, 0.8]	>0.1	<.001	**15.2***<br>[11.7, 18.8]**	**<0.0000<br>1**	**0.04**	1.4<br>[-0.6, 3.4]	>0.1	0.001	0.04
CST	-1.1<br>[-3.2, 1.0]	>0.1	<.001	**17.3***<br>[13.5, 21.1]**	**<0.0000<br>1**	**0.044**	0.4<br>[-1.7, 2.5]	>0.1	<.001	0.044
ML	-1.1<br>[-3.2, 1.0]	>0.1	<.001	**13.5***<br>[9.8, 17.3]**	**<0.0000<br>1**	**0.029**	1.2<br>[-0.8, 3.3]	>0.1	<.001	0.029

SCP	-0.9*<br>[-1.8, -0.1]	0.0247	0.003	3.3***<br>[1.8, 4.8]	<0.0000<br>1	0.011	-0.3<br>[-1.1, 0.5]	>0.1	<.001	0.014
ICP	-1.4*<br>[-2.6, -0.1]	0.0349	0.003	7.0***<br>[4.7, 9.2]	<0.0000<br>1	0.021	0.3<br>[-1.0, 1.6]	>0.1	<.001	0.022
Projection										
ACR	-2.3***<br>[-3.0, -1.5]	<0.00001	0.02	1.1<br>[-0.2, 2.5]	>0.1	0.002	0.3<br>[-0.5, 1.0]	>0.1	<.001	0.023
SCR	-0.6*<br>[-1.2, -0.0]	0.0489	0.002	-1.8**<br>[-2.9, -0.7]	0.0009	0.006	0.2<br>[-0.3, 0.8]	>0.1	<.001	0.007
PCR	-1.2*<br>[-2.1, -0.4]	0.0039	0.005	-5.0***<br>[-6.5, -3.5]	<0.0000<br>1	0.024	0.4<br>[-0.5, 1.2]	>0.1	<.001	0.026
ALIC	-1.6***<br>[-2.1, -1.0]	<0.00001	0.018	4.3***<br>[3.3, 5.3]	<0.0000<br>1	0.041	0.1<br>[-0.4, 0.7]	>0.1	<.001	0.063
PLIC	-0.5<br>[-1.0, 0.1]	0.0867	0.002	3.2***<br>[2.2, 4.1]	<0.0000<br>1	0.025	0.1<br>[-0.4, 0.6]	>0.1	<.001	0.026
RLIC	-1.5***<br>[-2.1, -0.8]	<0.00001	0.011	0.0<br>[-1.2, 1.2]	>0.1	<.001	0.2<br>[-0.5, 0.8]	>0.1	<.001	0.011
PTR	-1.0*<br>[-1.9, -0.2]	0.0118	0.004	-3.3***<br>[-4.8, -1.8]	<0.0000<br>1	0.011	0.6<br>[-0.3, 1.4]	>0.1	0.001	0.012
CP	-0.4<br>[-2.2, 1.4]	>0.1	<.001	13.9***<br>[10.7, 17.2]	<0.0000<br>1	0.04	-0.6<br>[-2.4, 1.2]	>0.1	<.001	0.039
Association										
FX	-0.1<br>[-1.8, 1.6]	>0.1	<.001	-4.4*<br>[-7.5, -1.3]	0.0059	0.004	-0.4<br>[-2.1, 1.4]	>0.1	<.001	0.003
FX/ST	-1.7***<br>[-2.4, -1.0]	<0.00001	0.013	-1.4*<br>[-2.7, -0.1]	0.035	0.003	0.3<br>[-0.4, 1.0]	>0.1	<.001	0.014
CgC	-2.7***<br>[-3.5, -2.0]	<0.00001	0.029	0.6<br>[-0.7, 2.0]	>0.1	<.001	0.3<br>[-0.5, 1.0]	>0.1	<.001	0.032
CgH	-2.8***<br>[-3.6, -1.9]	<0.00001	0.023	1.8*<br>[0.2, 3.3]	0.0248	0.003	0.3<br>[-0.5, 1.2]	>0.1	<.001	0.027
SFO	-1.3*<br>[-2.1, -0.4]	0.0028	0.005	-0.6<br>[-2.1, 0.9]	>0.1	<.001	-0.1<br>[-1.0, 0.7]	>0.1	<.001	0.005
SLF	-1.5***<br>[-2.2, -0.9]	<0.00001	0.012	-1.6*<br>[-2.8, -0.5]	0.0061	0.004	0.2<br>[-0.5, 0.8]	>0.1	<.001	0.015
EC	-1.9***<br>[-2.4, -1.3]	<0.00001	0.024	2.2***<br>[1.2, 3.3]	<0.0000<br>1	0.011	-0.0<br>[-0.6, 0.5]	>0.1	<.001	0.04
UNC	-1.7**<br>[-2.6, -0.8]	0.0003	0.008	0.8<br>[-0.9, 2.4]	>0.1	<.001	-0.0<br>[-1.0, 0.9]	>0.1	<.001	0.008
SS	-2.4***<br>[-3.1, -1.6]	<0.00001	0.02	-4.0***<br>[-5.4, -2.6]	<0.0000<br>1	0.017	0.7<br>[-0.1, 1.5]	0.0831	0.002	0.034
Commissural										

GCC	**-2.1*** [-2.9, -1.3]**	**<0.00001**	**0.014**	0.7 [-0.8, 2.1]	>0.1	<.001	0.5 [-0.4, 1.3]	>0.1	<.001	0.014
BCC	-1.2** [-2.0, -0.5]	0.0008	0.007	-2.4** [-3.7, -1.1]	0.0003	0.007	0.7 [-0.0, 1.4]	0.0507	0.002	0.012
SCC	**-1.7*** [-2.4, -1.0]**	**<0.00001**	**0.014**	**-2.4** [-3.6, -1.2]**	**0.0001**	**0.009**	0.3 [-0.4, 1.0]	>0.1	<.001	0.021
TAP	-2.4* [-3.9, -0.8]	0.0024	0.005	**-5.6*** [-8.4, -2.9]**	**0.0001**	**0.009**	0.6 [-0.9, 2.2]	>0.1	<.001	0.012

Supplemental Table 6. Model results for mean NDI in JHU ROIs.
The raw parameter estimate (β) values and their 95 % confidence intervals (in square brackets) are $x10^{-3}$/year change in NDI for Age and Age x Sex effects, and $x10^{-3}$ difference for Sex effect. The first columns give the abbreviated JHU ROIs (see Table 1 of the main text for their full names). Statistical significance symbols (uncorrected for multiple comparisons) *: 0.05 < p <0.001, **: 0.001 < p < 0.0001, ***: p < 0.0001. Bold symbols indicate Bonferroni-corrected significant p-values.

	Age			Sex			Age X Sex			
	β [95%CI]	*p value*	*η^2_G*	*β [95%CI]*	*p value*	*η^2_G*	*β [95%CI]*	*p value*	*η^2_G*	*adj. R2*
Brainstem										
MCP	1.1* [0.2, 1.9]	0.0109	0.003	**-16.2*** [-17.7, -14.8]**	**<0.00001**	**0.214**	-0.2 [-1.0, 0.7]	>0.1	<.001	0.219
PCT	0.3 [-0.7, 1.2]	>0.1	<.001	**-13.1*** [-14.8, -11.4]**	**<0.00001**	**0.116**	-0.0 [-1.0, 0.9]	>0.1	<.001	0.116
CST	0.2 [-1.1, 1.5]	>0.1	<.001	**-18.6*** [-21.0, -16.2]**	**<0.00001**	**0.123**	-0.1 [-1.4, 1.2]	>0.1	<.001	0.122
ML	2.2* [0.6, 3.7]	0.0056	0.003	**-41.1*** [-43.9, -38.3]**	**<0.00001**	**0.328**	-1.4 [-2.9, 0.2]	0.0805	0.001	0.334
SCP	**2.0*** [1.1, 2.9]**	**<0.00001**	**0.007**	**-25.5*** [-27.2, -23.9]**	**<0.00001**	**0.337**	-0.8 [-1.7, 0.1]	0.0989	0.001	0.348
ICP	**2.4*** [1.2, 3.5]**	**<0.00001**	**0.007**	**-25.8*** [-27.9, -23.8]**	**<0.00001**	**0.259**	-1.1 [-2.3, 0.0]	0.0582	0.002	0.27
Projection										
ACR	**2.9*** [1.9, 4.0]**	**<0.00001**	**0.016**	-2.6* [-4.6, -0.7]	0.0082	0.004	-0.1 [-1.1, 1.0]	>0.1	<.001	0.022
SCR	**2.2*** [1.3, 3.1]**	**<0.00001**	**0.012**	**-4.9*** [-6.6, -3.2]**	**<0.00001**	**0.019**	0.0 [-0.9, 1.0]	>0.1	<.001	0.033
PCR	**2.8*** [1.7, 3.9]**	**<0.00001**	**0.015**	**-6.0*** [-7.9, -4.1]**	**<0.00001**	**0.021**	-0.9 [-1.9, 0.2]	>0.1	0.001	0.036
ALIC	**3.1*** [2.1, 4.1]**	**<0.00001**	**0.02**	**-8.7*** [-10.5, -6.9]**	**<0.00001**	**0.049**	0.1 [-0.9, 1.1]	>0.1	<.001	0.074
PLIC	**1.8** [0.9, 2.8]**	**0.0002**	**0.007**	**-17.0*** [-18.8, -15.3]**	**<0.00001**	**0.18**	-0.1 [-1.1, 0.8]	>0.1	<.001	0.19

RLIC	**3.4***<br>[2.3, 4.5]**	**<0.00001**	**0.018**	**-18.7***<br>[-20.7, -16.7]**	**<0.00001**	**0.162**	-1.1<br>[-2.2, 0.0]	0.0612	0.002	0.184
PTR	**2.1***<br>[1.1, 3.1]**	**<0.00001**	**0.01**	**-3.5**<br>[-5.3, -1.7]**	**0.0002**	**0.008**	-1.3*<br>[-2.3, -0.3]	0.0139	0.003	0.018
CP	1.4<br>[-0.3, 3.1]	>0.1	0.001	**-24.8***<br>[-27.8, -21.8]**	**<0.00001**	**0.13**	0.3<br>[-1.4, 2.0]	>0.1	<.001	0.132
Association										
FX	**3.5***<br>[1.9, 5.1]**	**<0.00001**	**0.01**	**-12.2***<br>[-15.2, -9.3]**	**<0.00001**	**0.037**	0.7<br>[-1.0, 2.3]	>0.1	<.001	0.051
FX/ST	**3.7***<br>[2.5, 4.8]**	**<0.00001**	**0.018**	**-21.8***<br>[-23.9, -19.7]**	**<0.00001**	**0.192**	-0.8<br>[-1.9, 0.4]	>0.1	<.001	0.214
CgC	**3.8***<br>[2.7, 4.9]**	**<0.00001**	**0.021**	**-18.1***<br>[-20.1, -16.1]**	**<0.00001**	**0.151**	0.0<br>[-1.1, 1.2]	>0.1	<.001	0.18
CgH	**6.6***<br>[4.9, 8.3]**	**<0.00001**	**0.026**	**-34.9***<br>[-38.0, -31.9]**	**<0.00001**	**0.224**	-1.9*<br>[-3.5, -0.2]	0.0297	0.002	0.257
SFO	**3.5***<br>[2.2, 4.7]**	**<0.00001**	**0.016**	**-8.7***<br>[-11.0, -6.5]**	**<0.00001**	**0.032**	-0.1<br>[-1.3, 1.2]	>0.1	<.001	0.052
SLF	**2.6***<br>[1.8, 3.5]**	**<0.00001**	**0.019**	**-3.3***<br>[-4.9, -1.7]**	**0.0001**	**0.009**	-0.2<br>[-1.1, 0.7]	>0.1	<.001	0.031
EC	**3.3***<br>[2.5, 4.2]**	**<0.00001**	**0.028**	**-10.0***<br>[-11.7, -8.4]**	**<0.00001**	**0.078**	-0.4<br>[-1.3, 0.5]	>0.1	<.001	0.112
UNC	**4.1***<br>[2.9, 5.2]**	**<0.00001**	**0.025**	**-8.5***<br>[-10.6, -6.3]**	**<0.00001**	**0.034**	-0.9<br>[-2.1, 0.2]	>0.1	0.001	0.062
SS	**3.9***<br>[2.7, 5.1]**	**<0.00001**	**0.021**	**-10.3***<br>[-12.5, -8.1]**	**<0.00001**	**0.047**	-1.5*<br>[-2.7, -0.3]	0.0124	0.003	0.071
Commissural										
GCC	**2.4***<br>[1.2, 3.6]**	**0.0001**	**0.009**	-3.5*<br>[-5.7, -1.4]	0.0013	0.006	-0.1<br>[-1.3, 1.0]	>0.1	<.001	0.015
BCC	**2.4***<br>[1.4, 3.5]**	**<0.00001**	**0.011**	**-10.8***<br>[-12.7, -8.9]**	**<0.00001**	**0.068**	-0.2<br>[-1.3, 0.8]	>0.1	<.001	0.082
SCC	**2.6***<br>[1.6, 3.6]**	**<0.00001**	**0.013**	**-14.4***<br>[-16.2, -12.5]**	**<0.00001**	**0.121**	-0.9<br>[-1.9, 0.1]	0.0834	0.002	0.137
TAP	2.3*<br>[0.9, 3.8]	0.0016	0.006	-1.3<br>[-3.9, 1.3]	>0.1	<.001	-1.3<br>[-2.7, 0.2]	0.0891	0.002	0.005

Supplemental Table 7. Model results for mean ODI in JHU ROIs.
The raw parameter estimate (β) values and their 95 % confidence intervals (in square brackets) are $x10^{-3}$/year change in ODI for Age and Age x Sex effects, and $x10^{-3}$ difference in ODI for Sex effect. The first columns give the abbreviated JHU ROIs (see Table 1 of the main text for their full names). Statistical significance symbols (uncorrected for multiple comparisons) *: $0.05 < p < 0.001$, **: $0.001 < p < 0.0001$, ***: $p < 0.0001$. Bold symbols indicate Bonferroni-corrected significant p-values.

	Age			Sex			Age X Sex			*adj. R2*
	β [95%CI]	*p value*	η^2_G	*β [95%CI]*	*p value*	η^2_G	*β [95%CI]*	*p value*	η^2_G	
Brainstem										
MCP	0.7*<br>[0.1, 1.4]	0.0195	0.003	**-9.2***<br>[-10.3, -8.0]**	**<0.00001**	**0.132**	-0.3<br>[-0.9, 0.3]	>0.1	<.001	0.136
PCT	1.3*<br>[0.2, 2.4]	0.0165	0.003	**-21.4***<br>[-23.4, -19.5]**	**<0.00001**	**0.21**	-0.5<br>[-1.6, 0.6]	>0.1	<.001	0.214
CST	1.9*<br>[0.6, 3.2]	0.0047	0.004	**-13.5***<br>[-15.8, -11.1]**	**<0.00001**	**0.067**	-1.5*<br>[-2.9, -0.2]	0.0227	0.003	0.073
ML	1.3<br>[-0.0, 2.6]	0.0558	0.002	**-11.1***<br>[-13.5, -8.7]**	**<0.00001**	**0.047**	-0.4<br>[-1.7, 0.9]	>0.1	<.001	0.049
SCP	0.9*<br>[0.3, 1.5]	0.0041	0.004	**-10.6***<br>[-11.7, -9.5]**	**<0.00001**	**0.163**	-1.1**<br>[-1.7, -0.4]	0.0008	0.005	0.173
ICP	1.1*<br>[0.2, 2.1]	0.0197	0.003	**-9.2***<br>[-10.9, -7.5]**	**<0.00001**	**0.063**	-0.8<br>[-1.7, 0.2]	>0.1	0.001	0.066
Projection										
ACR	-0.2<br>[-0.8, 0.4]	>0.1	<.001	-0.8<br>[-1.9, 0.3]	>0.1	0.001	-0.1<br>[-0.7, 0.5]	>0.1	<.001	<0.00001
SCR	**1.2***<br>[0.6, 1.8]**	**<0.00001**	**0.01**	-1.6*<br>[-2.7, -0.6]	0.0025	0.005	-0.1<br>[-0.7, 0.5]	>0.1	<.001	0.015
PCR	0.4<br>[-0.2, 1.0]	>0.1	0.001	**-3.4***<br>[-4.6, -2.3]**	**<0.00001**	**0.021**	-0.5<br>[-1.1, 0.1]	0.0947	0.002	0.022
ALIC	0.7*<br>[0.2, 1.1]	0.0061	0.004	-0.7<br>[-1.5, 0.2]	>0.1	0.001	-0.2<br>[-0.7, 0.3]	>0.1	<.001	0.004
PLIC	**1.3***<br>[0.8, 1.7]**	**<0.00001**	**0.018**	**-2.9***<br>[-3.7, -2.1]**	**<0.00001**	**0.03**	-0.5*<br>[-0.9, -0.0]	0.0384	0.002	0.049
RLIC	0.5*<br>[0.1, 0.9]	0.0095	0.003	**-6.9***<br>[-7.5, -6.2]**	**<0.00001**	**0.184**	-0.6*<br>[-0.9, -0.2]	0.0044	0.004	0.192
PTR	0.5*<br>[0.1, 0.8]	0.0115	0.004	**-2.5***<br>[-3.2, -1.8]**	**<0.00001**	**0.031**	-0.3<br>[-0.6, 0.1]	>0.1	0.001	0.035
CP	**2.4***<br>[1.6, 3.2]**	**<0.00001**	**0.019**	**-4.3***<br>[-5.8, -2.9]**	**<0.00001**	**0.019**	-1.2*<br>[-2.0, -0.4]	0.0036	0.005	0.039
Association										
FX	1.4*<br>[0.3, 2.4]	0.0103	0.004	**-9.0***<br>[-10.9, -7.1]**	**<0.00001**	**0.048**	-0.7<br>[-1.8, 0.3]	>0.1	<.001	0.052
FX/ST	0.8*<br>[0.2, 1.4]	0.0103	0.003	**-7.7***<br>[-8.8, -6.7]**	**<0.00001**	**0.106**	-0.7*<br>[-1.3, -0.1]	0.0235	0.003	0.112
CgC	-0.5<br>[-1.2, 0.1]	>0.1	0.001	**-5.0***<br>[-6.1, -3.8]**	**<0.00001**	**0.038**	0.2<br>[-0.5, 0.8]	>0.1	<.001	0.037
CgH	1.6**<br>[0.7, 2.4]	0.0003	0.006	**-16.6***<br>[-18.1, -15.1]**	**<0.00001**	**0.213**	-1.2*<br>[-2.0, -0.3]	0.0056	0.004	0.223

SFO	0.6 [-0.2, 1.5]	>0.1	0.001	**-5.1*** [-6.6, -3.6]**	**<0.00001**	**0.025**	-0.6 [-1.4, 0.2]	>0.1	0.001	0.026
SLF	-0.1 [-0.6, 0.3]	>0.1	<.001	0.0 [-0.8, 0.8]	>0.1	<.001	-0.2 [-0.7, 0.2]	>0.1	<.001	−0.001
EC	0.3 [-0.2, 0.7]	>0.1	<.001	**-2.1*** [-2.8, -1.3]**	**<0.00001**	**0.016**	-0.1 [-0.5, 0.3]	>0.1	<.001	0.016
UNC	0.8* [0.2, 1.3]	0.0065	0.004	**-3.4*** [-4.3, -2.4]**	**<0.00001**	**0.026**	-0.2 [-0.8, 0.3]	>0.1	<.001	0.03
SS	-0.0 [-0.4, 0.4]	>0.1	<.001	**-5.9*** [-6.6, -5.2]**	**<0.00001**	**0.13**	-0.3 [-0.7, 0.1]	>0.1	<.001	0.13
Commissural										
GCC	-0.4 [-0.9, 0.1]	0.0927	0.002	-1.5** [-2.3, -0.6]	0.0009	0.006	0.2 [-0.3, 0.6]	>0.1	<.001	0.006
BCC	0.3 [-0.1, 0.7]	>0.1	<.001	**-2.7*** [-3.5, -2.0]**	**<0.00001**	**0.029**	0.1 [-0.3, 0.5]	>0.1	<.001	0.03
SCC	0.1 [-0.3, 0.5]	>0.1	<.001	**-6.4*** [-7.1, -5.7]**	**<0.00001**	**0.158**	-0.4* [-0.8, -0.0]	0.0299	0.002	0.16
TAP	-0.3 [-1.2, 0.6]	>0.1	<.001	-0.0 [-1.7, 1.7]	>0.1	<.001	-0.5 [-1.4, 0.4]	>0.1	<.001	−0.000

Supplemental Table 8. Model results for mean IsoVF in JHU ROIs.
The raw parameter estimate (*β*) values and their 95 % confidence intervals (in square brackets) are $x10^{-3}$/year change in IsoVF for Age and Age x Sex effects, and $x10^{-3}$ difference in IsoVF for Sex effect. The first columns give the abbreviated JHU ROIs (see Table 1 of the main text for their full names). Statistical significance symbols (uncorrected for multiple comparisons) *: $0.05 < p < 0.001$, **: $0.001 < p < 0.0001$, ***: $p < 0.0001$. Bold symbols indicate Bonferroni-corrected significant *p*-values.

	Age			Sex			Age X Sex			
	β [95%CI]	*p value*	η^2_G	*β [95%CI]*	*p value*	η^2_G	*β [95%CI]*	*p value*	η^2_G	*adj. R2*
Brainstem										
MCP	-0.6* [-1.1, -0.0]	0.0365	0.002	**7.5*** [6.6, 8.5]**	**<0.00001**	**0.124**	0.6* [0.1, 1.2]	0.018	0.003	0.129
PCT	-1.9* [-3.5, -0.3]	0.0229	0.003	**25.7*** [22.8, 28.6]**	**<0.00001**	**0.147**	1.8* [0.2, 3.4]	0.0302	0.002	0.152
CST	-2.0* [-3.6, -0.3]	0.0172	0.003	**23.2*** [20.3, 26.1]**	**<0.00001**	**0.126**	1.3 [-0.3, 2.9]	>0.1	0.001	0.13
ML	-1.5* [-2.9, -0.0]	0.0429	0.002	**7.6*** [5.1, 10.2]**	**<0.00001**	**0.02**	1.3 [-0.1, 2.7]	0.0731	0.002	0.022
SCP	**-1.2*** [-1.8, -0.6]**	**0.0001**	**0.009**	**6.3*** [5.2, 7.4]**	**<0.00001**	**0.068**	0.6 [-0.1, 1.2]	0.0731	0.002	0.078

ICP	-1.2*<br>[-2.2, -0.2]	0.0151	0.003	**3.4****<br>**[1.7, 5.2]**	**0.0001**	**0.009**	0.3<br>[-0.7, 1.3]	>0.1	<.001	0.011
Projection										
ACR	-0.1<br>[-0.5, 0.3]	>0.1	<.001	0.3<br>[-0.4, 1.0]	>0.1	<.001	0.4<br>[-0.0, 0.8]	0.0796	0.002	0.001
SCR	-0.1<br>[-0.4, 0.2]	>0.1	<.001	**-3.5*****<br>**[-4.1, -3.0]**	**<0.00001**	**0.077**	0.4*<br>[0.1, 0.7]	0.0096	0.004	0.078
PCR	0.5*<br>[0.1, 0.9]	0.0234	0.003	**-6.8*****<br>**[-7.6, -6.1]**	**<0.00001**	**0.147**	0.1<br>[-0.3, 0.5]	>0.1	<.001	0.151
ALIC	-0.4<br>[-0.8, 0.0]	0.0678	0.002	0.3<br>[-0.4, 1.1]	>0.1	<.001	0.4<br>[-0.0, 0.8]	0.0572	0.002	0.002
PLIC	-0.6**<br>[-1.0, -0.3]	0.0007	0.007	-1.0*<br>[-1.6, -0.3]	0.0028	0.005	0.5*<br>[0.1, 0.9]	0.0076	0.004	0.011
RLIC	-0.1<br>[-0.5, 0.3]	>0.1	<.001	**-3.8*****<br>**[-4.5, -3.0]**	**<0.00001**	**0.057**	0.1<br>[-0.3, 0.5]	>0.1	<.001	0.056
PTR	0.1<br>[-0.3, 0.4]	>0.1	<.001	**-3.4*****<br>**[-4.1, -2.7]**	**<0.00001**	**0.051**	-0.1<br>[-0.5, 0.3]	>0.1	<.001	0.05
CP	-1.6*<br>[-2.7, -0.4]	0.011	0.004	**10.8*****<br>**[8.7, 13.0]**	**<0.00001**	**0.054**	0.3<br>[-0.9, 1.5]	>0.1	<.001	0.058
Association										
FX	2.0*<br>[0.6, 3.4]	0.0055	0.005	-0.2<br>[-2.7, 2.4]	>0.1	<.001	0.2<br>[-1.3, 1.6]	>0.1	<.001	0.004
FX/ST	-0.6*<br>[-1.1, -0.1]	0.0209	0.003	**-4.9*****<br>**[-5.8, -3.9]**	**<0.00001**	**0.059**	0.4<br>[-0.1, 0.9]	>0.1	0.001	0.059
CgC	-0.3<br>[-0.8, 0.1]	>0.1	0.001	**-5.8*****<br>**[-6.6, -5.1]**	**<0.00001**	**0.114**	0.3<br>[-0.1, 0.8]	>0.1	0.001	0.113
CgH	-0.2<br>[-0.8, 0.4]	>0.1	<.001	**-6.9*****<br>**[-8.1, -5.8]**	**<0.00001**	**0.077**	0.2<br>[-0.4, 0.8]	>0.1	<.001	0.075
SFO	-0.1<br>[-0.6, 0.4]	>0.1	<.001	**-2.2*****<br>**[-3.1, -1.3]**	**<0.00001**	**0.014**	0.3<br>[-0.2, 0.8]	>0.1	<.001	0.013
SLF	0.1<br>[-0.2, 0.4]	>0.1	<.001	**-3.5*****<br>**[-4.0, -2.9]**	**<0.00001**	**0.087**	0.2<br>[-0.1, 0.5]	>0.1	<.001	0.088
EC	0.2<br>[-0.2, 0.6]	>0.1	<.001	**-2.5*****<br>**[-3.3, -1.7]**	**<0.00001**	**0.023**	-0.1<br>[-0.6, 0.3]	>0.1	<.001	0.023
UNC	0.7*<br>[0.1, 1.3]	0.0325	0.003	**-2.8*****<br>**[-3.9, -1.7]**	**<0.00001**	**0.014**	-0.3<br>[-0.9, 0.3]	>0.1	<.001	0.016
SS	0.1<br>[-0.3, 0.4]	>0.1	<.001	**-5.3*****<br>**[-6.0, -4.6]**	**<0.00001**	**0.115**	-0.0<br>[-0.4, 0.4]	>0.1	<.001	0.114
Commissural										
GCC	-0.3<br>[-0.7, 0.1]	>0.1	0.001	0.8*<br>[0.1, 1.6]	0.0326	0.003	0.4<br>[-0.0, 0.8]	0.073	0.002	0.003

BCC	-0.1<br>[-0.5, 0.3]	>0.1	<.001	**-4.7*****<br>**[-5.3, -4.0]**	**<0.00001**	**0.099**	0.6**<br>[0.3, 1.0]	0.001	0.006	0.102
SCC	-0.5*<br>[-0.8, -0.1]	0.0124	0.004	-0.8*<br>[-1.5, -0.2]	0.0126	0.004	0.5*<br>[0.1, 0.9]	0.0091	0.004	0.007
TAP	0.5<br>[-0.5, 1.4]	>0.1	<.001	-2.1*<br>[-3.8, -0.3]	0.0227	0.003	0.3<br>[-0.7, 1.3]	>0.1	<.001	0.002

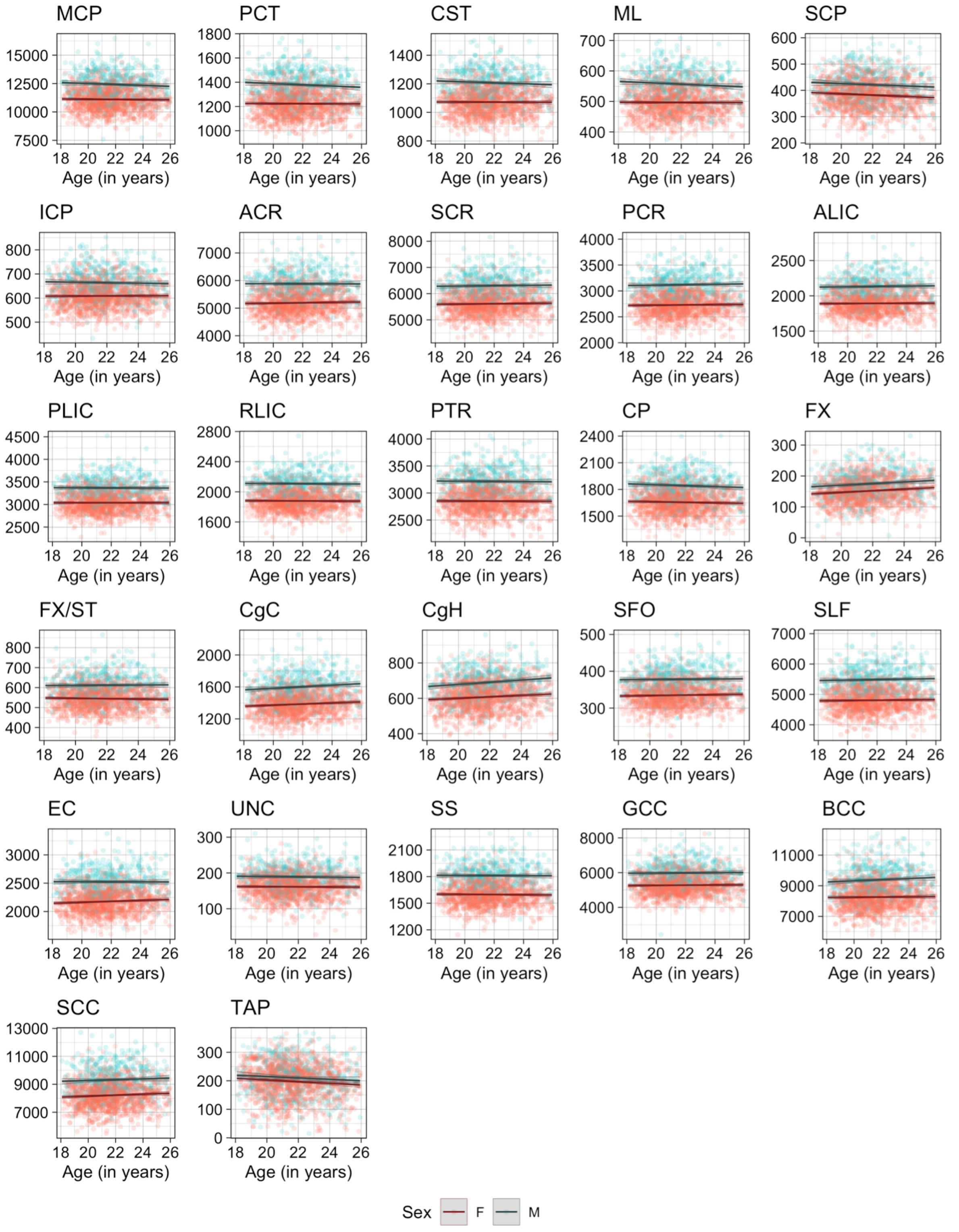

Supplemental Figure 5. Scatter plots of individual age effects on WM volumes (in mm^3) in each ROI.
Predicted linear regression lines are superimposed for each sex (dark red: females, dark cyan: males), with shades indicating the 95% confidence intervals. See Table 1 in the main text for the full names of the abbreviated ROIs.

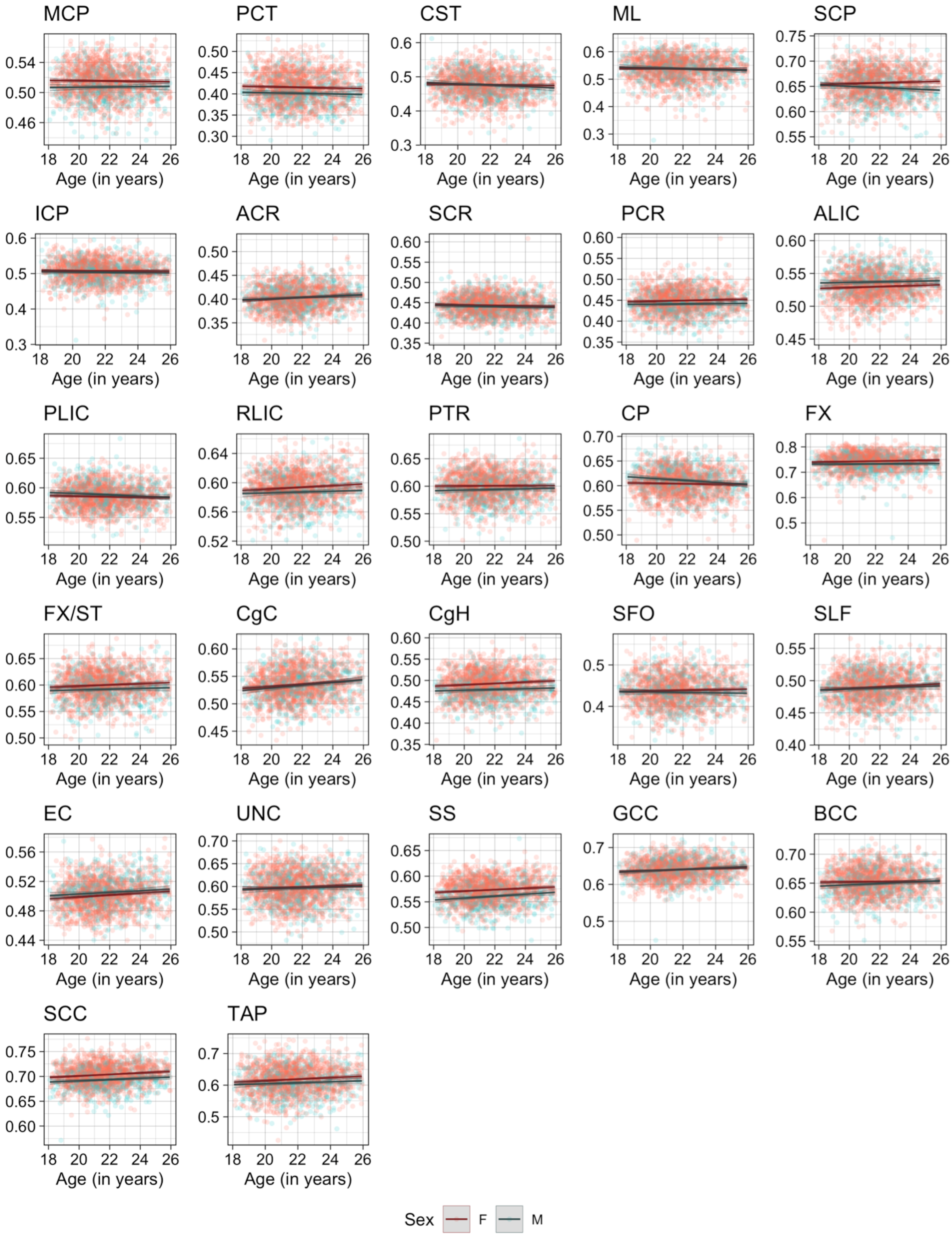

Supplemental Figure 6. Scatter plots of individual age effects on WM FA values in each ROI. Predicted linear regression lines are superimposed for each sex (dark red: females, dark cyan: males), with shades indicating the 95% confidence intervals. See Table 1 in the main text for the full names of the abbreviated ROIs.

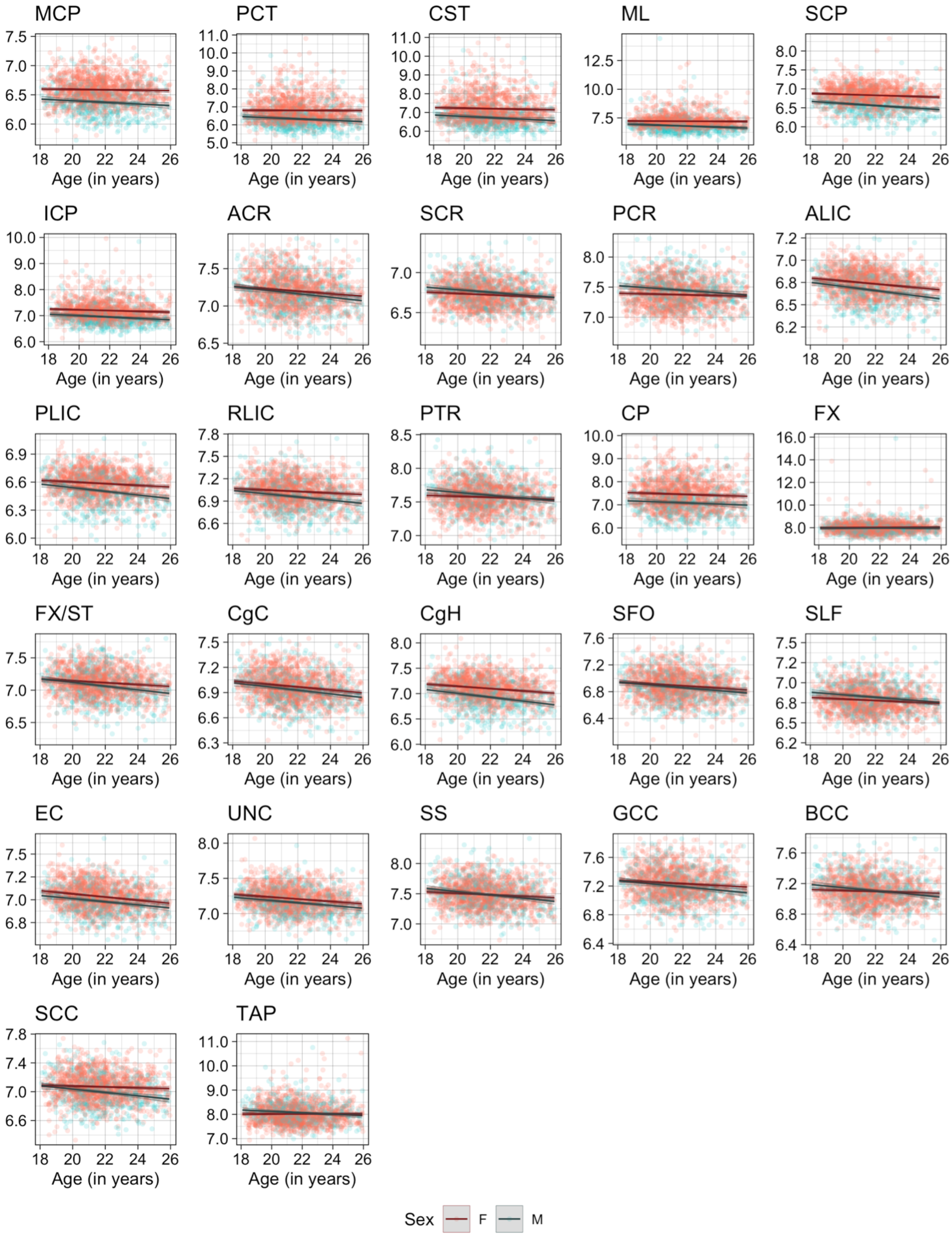

Supplemental Figure 7. Scatter plots of individual age effects on WM MD values (x10^{-4} mm^2/sec) in each ROI.
Predicted linear regression lines are superimposed for each sex (dark red: females, dark cyan: males), with shades indicating the 95% confidence intervals. See Table 1 in the main text for the full names of the abbreviated ROI.

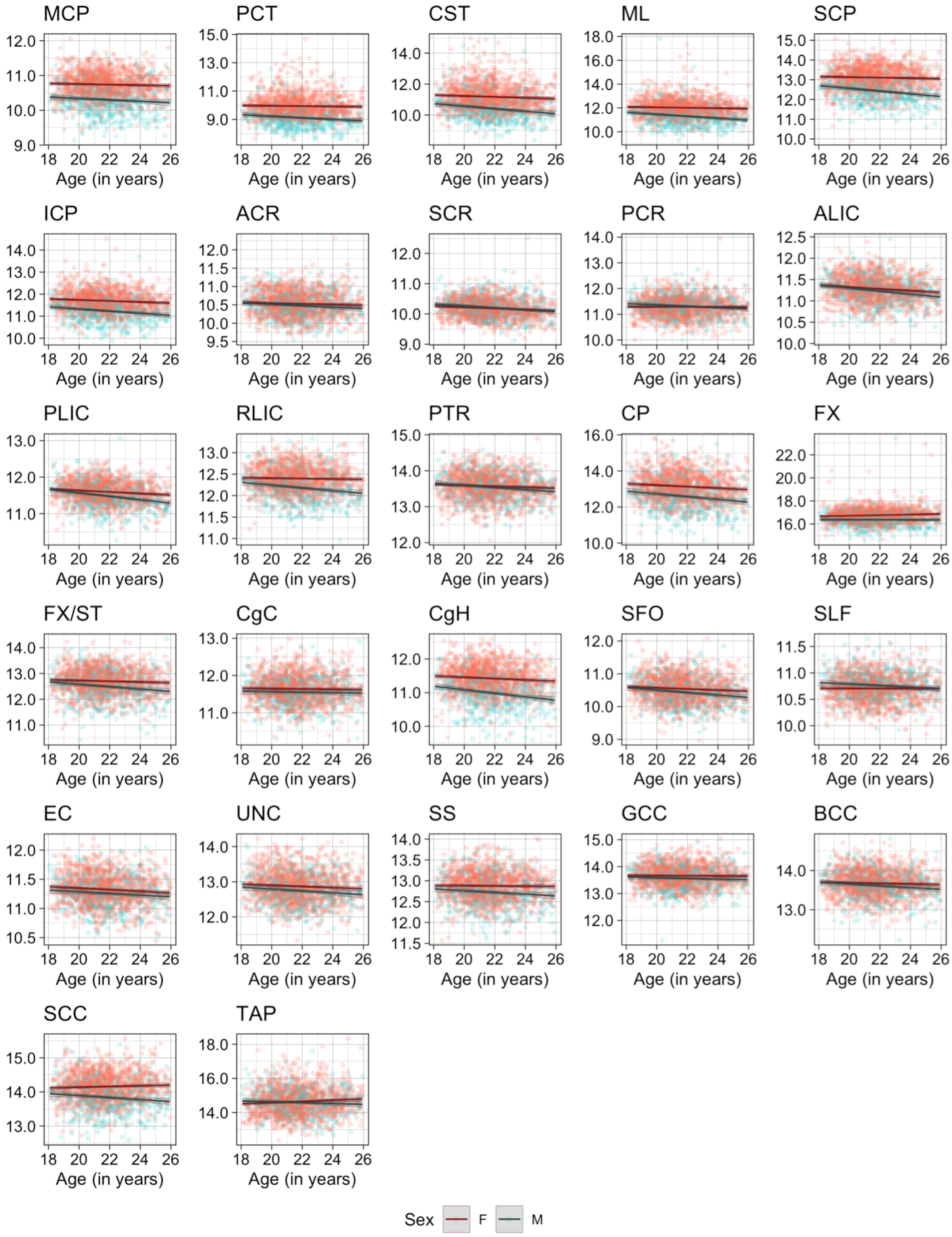

Supplemental Figure 8. Scatter plots of individual age effects on WM AD values (x10^{-4} mm^2/sec) in each ROI.

Predicted linear regression lines are superimposed for each sex (dark red: females, dark cyan: males), with shades indicating the 95% confidence intervals. See Table 1 in the main text for the full names of the abbreviated ROIs.

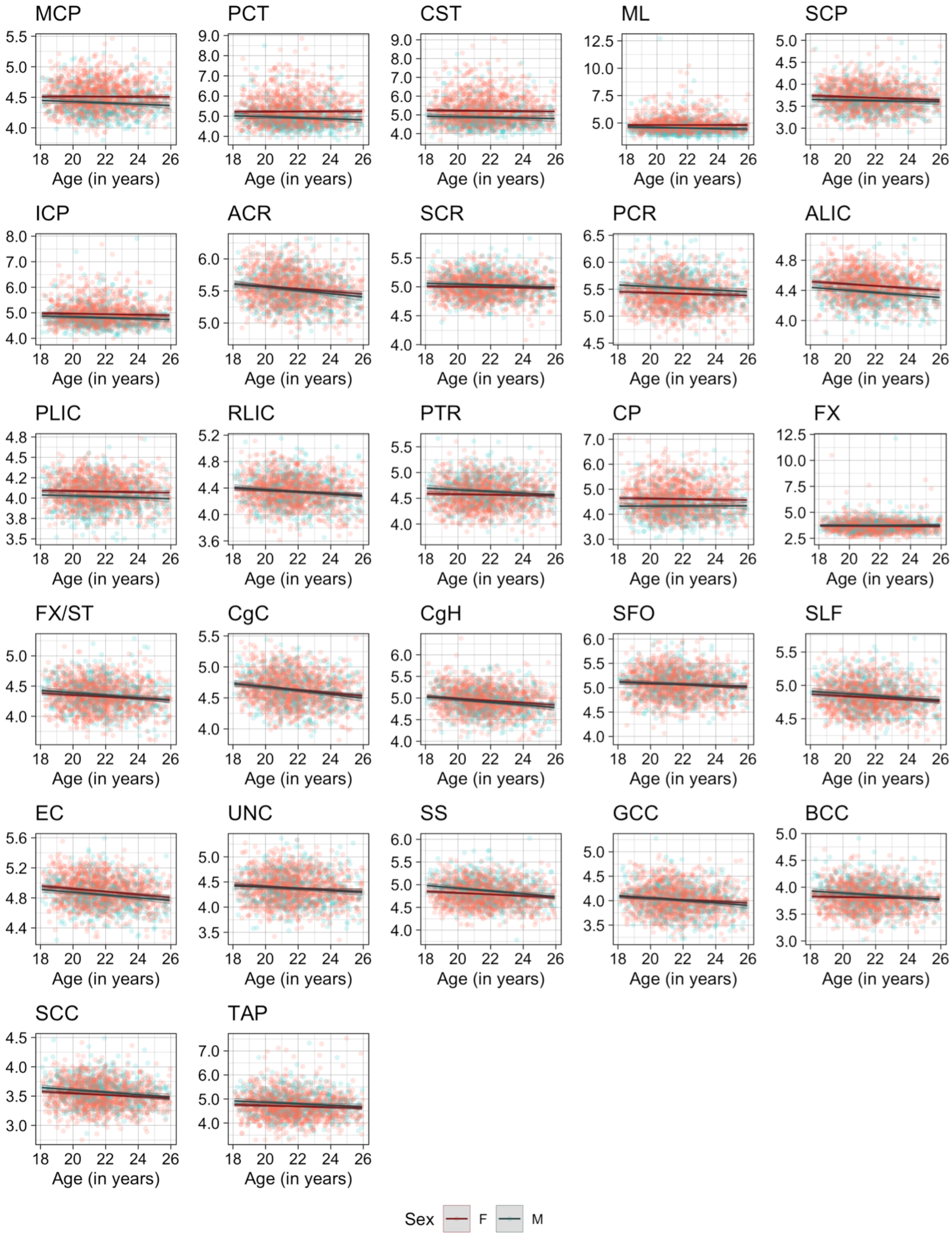

Supplemental Figure 9. Scatter plots of individual age effects on WM RD values (x10^{-4} mm^2/sec) in each ROI.
Predicted linear regression lines are superimposed for each sex (dark red: females, dark cyan: males), with shades indicating the 95% confidence intervals. See Table 1 in the main text for the full names of the abbreviated ROIs.

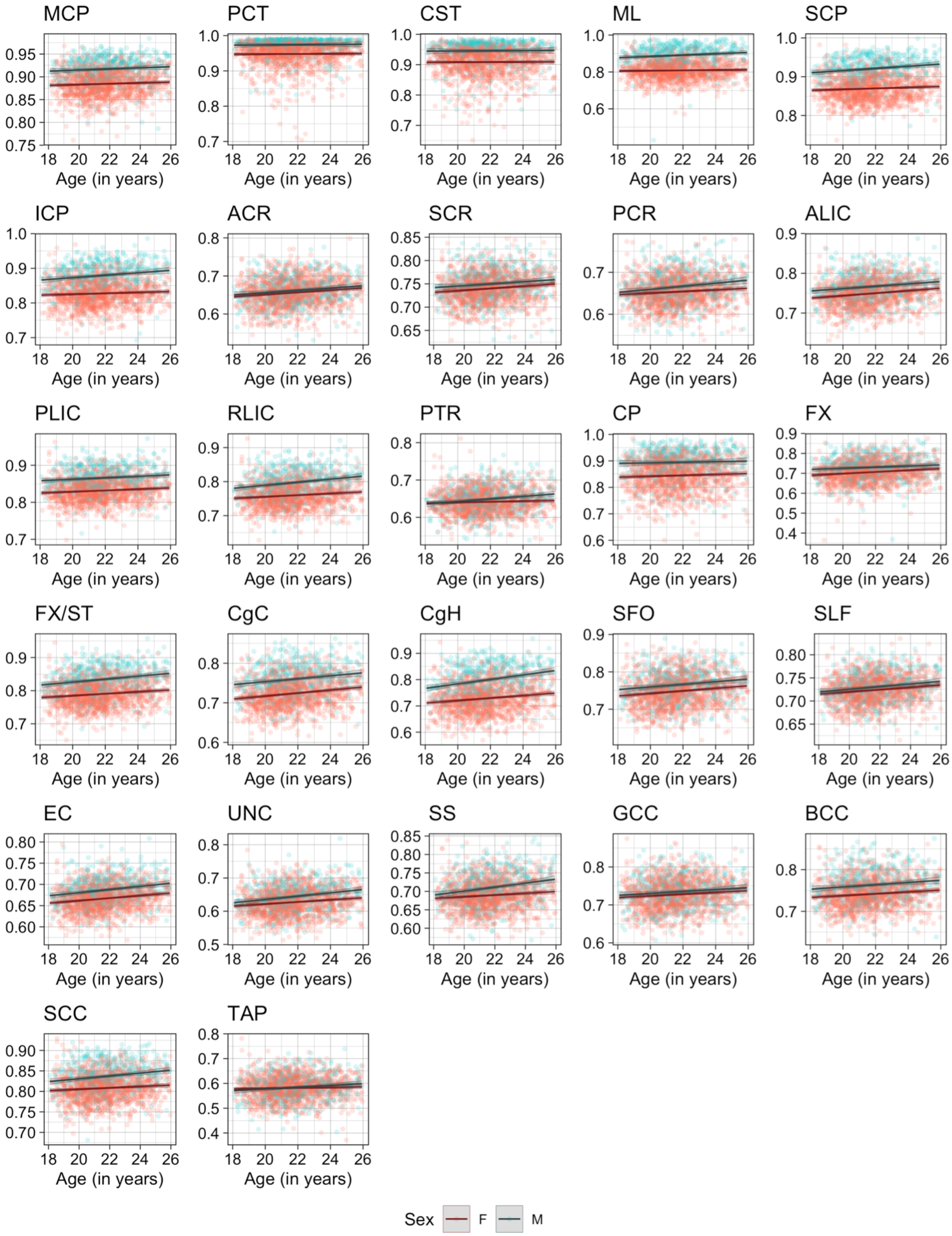

Supplemental Figure 10. Scatter plots of individual age effects on WM NDI values in each ROI. Predicted linear regression lines are superimposed for each sex (dark red: females, dark cyan: males), with shades indicating the 95% confidence intervals. See Table 1 in the main text for the full names of the abbreviated ROIs.

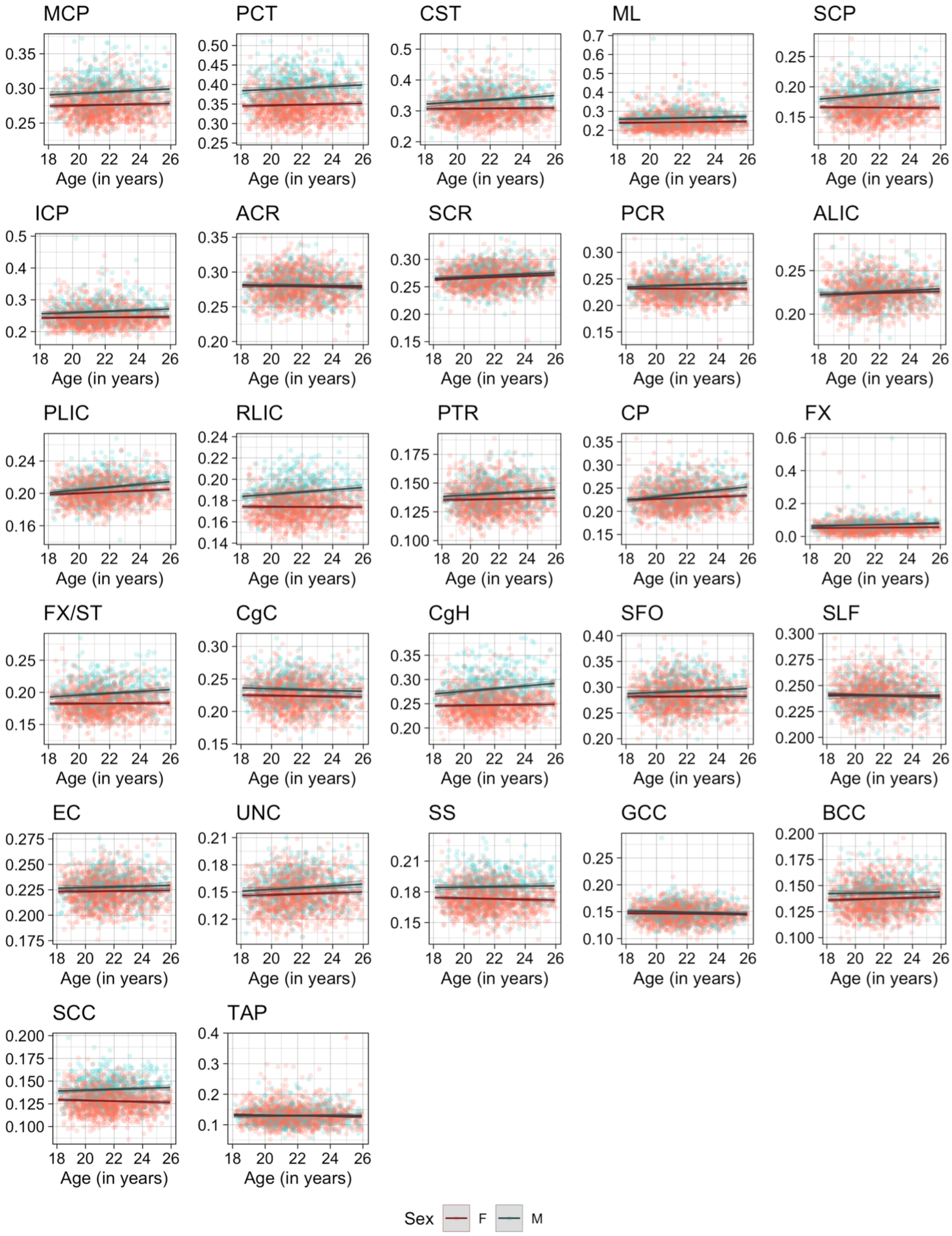

Supplemental Figure 11. Scatter plots of individual age effects on WM ODI values in each ROI. Predicted linear regression lines are superimposed for each sex (dark red: females, dark cyan: males), with shades indicating the 95% confidence intervals. See Table 1 in the main text for the full names of the abbreviated ROIs.

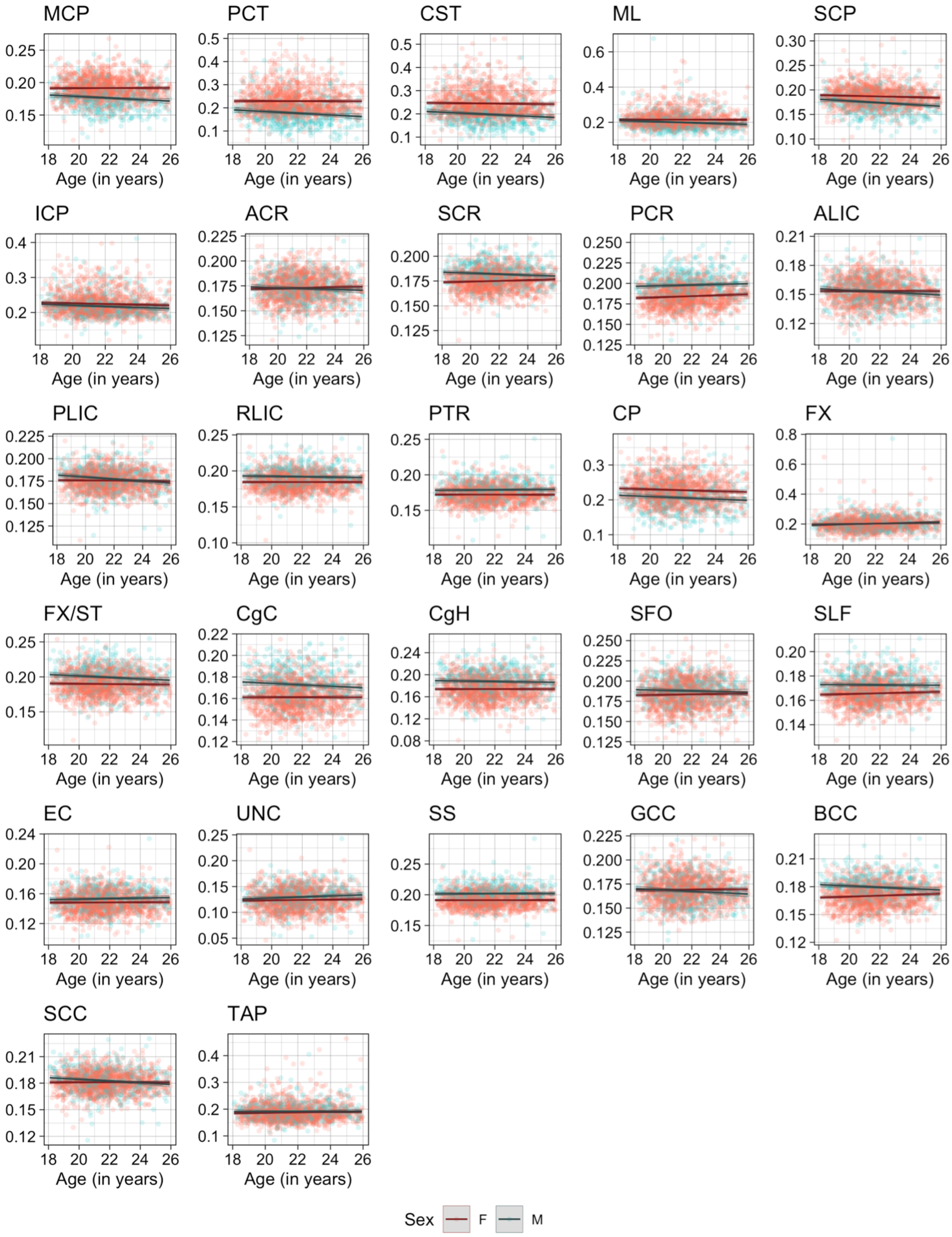

Supplemental Figure 12. Scatter plots of individual age effects on WM IsoVF values in each ROI.
Predicted linear regression lines are superimposed for each sex (dark red: females, dark cyan: males), with shades indicating the 95% confidence intervals. See Table 1 in the main text for the full names of the abbreviated ROIs.

4. Interrelations among the regional mean values of the WM metrics

In order to compare with the correlation structure of the regional age effects of the WM metrics, we computed a similar correlation matrix using the estimated mean values of each of the eight WM metrics (WM volume and mean DTI/NODDI values). We used the estimated mean values at mean age rather than actual mean values to account for the fact that raw mean values are dominated by female data, while the regional age effects represent those across two sexes. However, in practice, the correlation structure is almost identical when the actual or estimated mean values are used. We also standardised each metric across the ROIs to allow the comparison of metrics using the same scale.

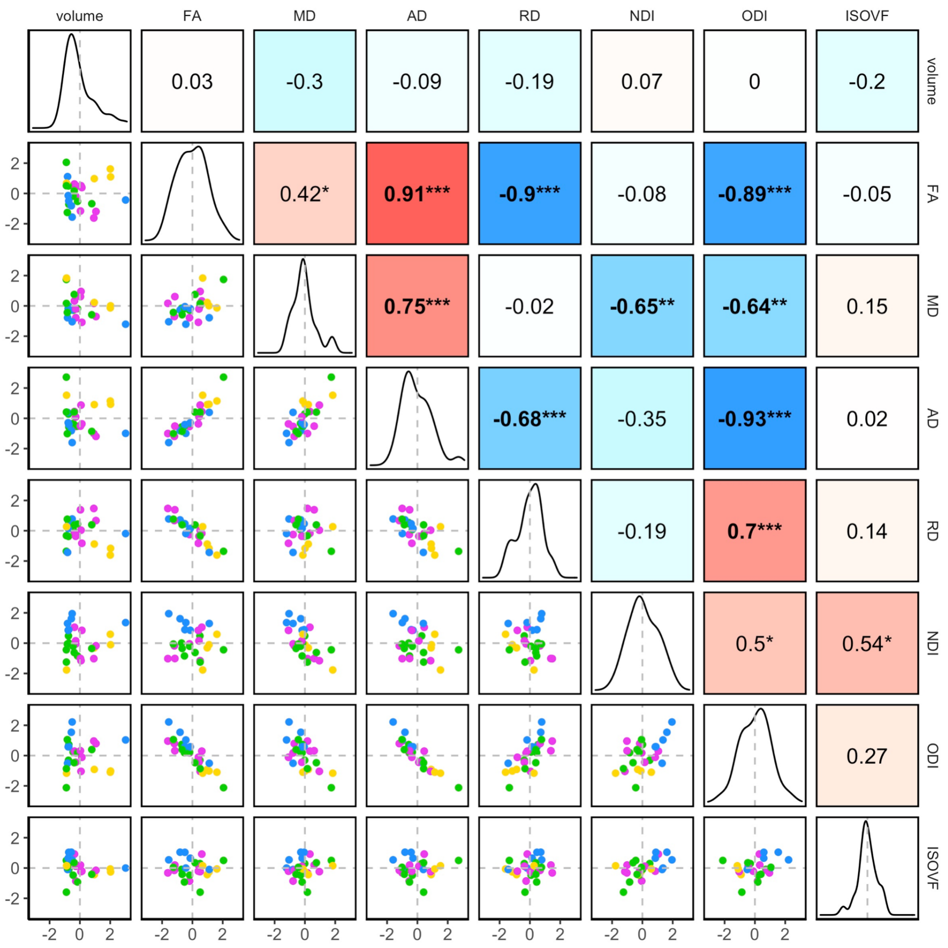

Supplemental Figure 13. The inter-relations between the regional mean values of the WM volume and microstructure phenotypes.
Pairwise correlations of the estimated mean values of the WM metrics in the 27 ROIs are shown. The diagonal of the plot matrix shows the distributions of the regional WM volume and DTI/NODDI values after standardising each metric across the ROIs. The upper triangle shows Pearson's correlation (*r*) values. The lower triangle shows the pairwise scatter plots of the standardised mean values, with the colours indicating the ROI groups (*blue*: brainstem, *pink*: projection, *green*: association, *yellow*: commissural). Statistical significance symbols (uncorrected for multiple comparisons) *: 0.05 < p <0.001, **: 0.001 < p < 0.0001, ***: p < 0.0001. Bold-face indicates a significant correlation after Bonferroni correction for multiple comparisons (28 correlations).

5. References

Bastiani, M., Cottaar, M., Fitzgibbon, S. P., Suri, S., Alfaro-Almagro, F., Sotiropoulos, S. N., Jbabdi, S., and Andersson, J. L. R. (2019). Automated quality control for within and between studies diffusion MRI data using a non-parametric framework for movement and distortion correction. *Neuroimage* 184, 801–812. doi:10.1016/j.neuroimage.2018.09.073.

Beaudet, G., Tsuchida, A., Petit, L., Tzourio, C., Caspers, S., Schreiber, J., Pausova, Z., Patel, Y., Paus, T., Schmidt, R., et al. (2020). Age-Related Changes of Peak Width Skeletonized Mean Diffusivity (PSMD) Across the Adult Lifespan: A Multi-Cohort Study. *Front. Psychiatry* 11, 342. doi:10.3389/fpsyt.2020.00342.

Cox, R. W. (1996). AFNI: software for analysis and visualization of functional magnetic resonance neuroimages. *Comput. Biomed. Res.* 29, 162–173. doi:10.1006/cbmr.1996.0014.

Dale, A. M., Fischl, B., and Sereno, M. I. (1999). Cortical surface-based analysis. I. Segmentation and surface reconstruction. *Neuroimage* 9, 179–194. doi:10.1006/nimg.1998.0395.

Pines, A. R., Cieslak, M., Larsen, B., Baum, G. L., Cook, P. A., Adebimpe, A., Dávila, D. G., Elliott, M. A., Jirsaraie, R., Murtha, K., et al. (2020). Leveraging multi-shell diffusion for studies of brain development in youth and young adulthood. *Dev. Cogn. Neurosci.* 43, 100788. doi:10.1016/j.dcn.2020.100788.

Rosen, A. F. G., Roalf, D. R., Ruparel, K., Blake, J., Seelaus, K., Villa, L. P., Ciric, R., Cook, P. A., Davatzikos, C., Elliott, M. A., et al. (2018). Quantitative assessment of structural image quality. *Neuroimage* 169, 407–418. doi:10.1016/j.neuroimage.2017.12.059.

Tournier, J.-D., Mori, S., and Leemans, A. (2011). Diffusion tensor imaging and beyond. *Magn. Reson. Med.* 65, 1532–1556. doi:10.1002/mrm.22924.

Tsuchida, A., Laurent, A., Crivello, F., Petit, L., Joliot, M., Pepe, A., Beguedou, N., Gueye, M.-F., Verrecchia, V., Nozais, V., et al. (2020). The MRi-Share database: brain imaging in a cross-sectional cohort of 1,870 university students. *BioRxiv*. doi:10.1101/2020.06.17.154666.

Tukey, J. W. (1977). *Exploratory Data Analysis*. 1st ed. Reading, Mass: Pearson.
